# Genomic epidemiology of *Histoplasma* in Africa

**DOI:** 10.1128/mbio.00564-25

**Published:** 2025-08-05

**Authors:** Rutendo E. Mapengo, Tsidiso G. Maphanga, Gaston I. Jofre, Jonathan A. Rader, David A. Turissini, Monica Birkhead, S. Ama Kwabia, Victoria E. Sepúlveda, Maria José Buitrago, Marcus de Melo Teixeira, Bridget M. Barker, Alexandre Alanio, Aude Sturny-Leclère, Dea Garcia-Hermoso, Nelesh P. Govender, Daniel R. Matute

**Affiliations:** 1 Wits Mycology Division, School of Pathology, Faculty of Health Sciences, University of the Witwatersrand 219580, Parktown, Gauteng, South Africa; 2 National Institute for Communicable Diseases, A Division of the National Health Laboratory Service 70687 , Johannesburg, Gauteng, South Africa; 3 Department of Biology, The University of North Carolina at Chapel Hill 169102, Chapel Hill, North Carolina, USA; 4 Department of Biology, Virginia Commonwealth University 172853, Richmond, Virginia, USA; 5 Department of Biology and Chemistry, Texas A&M International University, Laredo, Texas, USA; 6 Mycology Reference Laboratory, National Centre for Microbiology, Instituto de Salud Carlos III 38176, Madrid, Spain; 7 CIBERINFECT, ISCIII-CIBER de Enfermedades infecciosas, Instituto de Salud Carlos III 38176, Madrid, Spain; 8 Faculty of Medicine, University of Brasilia 468973, Brasília, Brazil; 9 Pathogen & Microbiome Institute, Northern Arizona University (NAU) 3356, Flagstaff, Arizona, USA; 10 Mycology-Parasitology Department, Institut Pasteur 27058, Paris, France; 11 Division of Medical Microbiology, Faculty of Health Sciences, University of Cape Town 37716, Rondebosch, Western Cape, South Africa; 12 Medical Research Council Centre for Medical Mycology at the University of Exeter 3286, Exeter, United Kingdom; 13 Institute of Infection and Immunity, City St George’s University of London 244882, London, United Kingdom; McMaster University, Hamilton, Ontario, Canada

**Keywords:** *Histoplasma*, speciation, introgression, selection, Africa

## Abstract

Histoplasmosis, the disease caused by thermally dimorphic fungi in the genus *Histoplasma*, is usually associated with pulmonary involvement in healthy individuals and a disseminated syndrome in immunocompromised patients. Among African patients, lung disease is less commonly reported than skin, lymph node, or bone disease. Because different species or strains may be associated with different disease presentations and outcomes, understanding genetic and phenotypic variation in the genus *Histoplasma* is important for accurate diagnosis and treatment. We sequenced the genomes of 36 *Histoplasma* isolates from Africa and used population genomics to measure the genetic variation of the genus on the African continent and to compare the genetic diversity of these isolates to the previously described Indian and American phylogenetic species. We found that strains from Africa belong to genetic lineages that are differentiated enough to be considered a phylogenetic species. The first, the *Africa* lineage, is consistent with a previously described species (*Histoplasma capsulatum duboisii*) which includes clinical cases more frequently associated with extrapulmonary manifestations than cases caused by other lineages. While there is some evidence of gene flow between *Histoplasma* lineages, it has not precluded divergence. A second lineage corresponding to *Histoplasma capsulatum farciminosum (Hcf*) includes all the isolates from equine samples. We identified loci under selection in these two better-sampled lineages and found loci that have undergone parallel positive selection. A single African isolate resembles a South American lineage. Finally, we measured the potential range expansion of the disease using climatic projections, highlighting the need to implement surveillance to monitor phylogenetic species of *Histoplasma* across Africa.

**IMPORTANCE:**

*Histoplasma* fungi, which cause histoplasmosis, are widespread and considered high-priority pathogens. While researchers have identified multiple genetically distinct lineages worldwide, little is known about *Histoplasma* diversity in Africa due to minimal sampling and inadequate diagnostics. Our study addresses this gap using population genomics to analyze stored African isolates. We identified three distinct groups: one of them is endemic to Africa and aligns with *Histoplasma capsulatum duboisii*, a lineage linked to skin-involved infections, while another lineage (*Hcf*) matches *Histoplasma capsulatum farciminosum*, associated with equine lymphangitis. Additionally, one African isolate closely resembles a South American lineage (mz5-like). These three lineages are genetically unique enough to be considered separate species. By integrating phylogenetics, clinical data, and environmental modeling, we provide the most comprehensive genetic assessment of African *Histoplasma* to date. This work not only enhances our understanding of an overlooked pathogen but also offers a model for studying other neglected fungi with global health implications.

## INTRODUCTION

Histoplasmosis, a disease caused by fungi in the genus *Histoplasma*, has a global range (1–3), and *Histoplasma* spp. are considered high-priority fungal pathogens (4, 5). Among immunocompromised patients, histoplasmosis can progress to disseminated disease and is frequently fatal (6–8). Substantial progress has been made to understand the molecular mechanisms of pathogenesis and evolutionary processes in a variety of *Histoplasma* spp. (reviewed in references 8 and 9). Histoplasmosis is a systemic mycosis with an incidence that can range from relatively low in temperate climates (0.1–1.0 case per 100,000 inhabitants per year) to up to more than 100 cases per 100,000 in high-risk groups and during epidemics (8, 10, 11).

Despite this progress, the causal agents of African histoplasmosis remain largely understudied. This is a gap because *Histoplasma* is presumed to have a wide range across the continent (12), and the outcome of histoplasmosis in African patients is often poor (13, 14). Nonetheless, the disease burden remains largely unknown (15, 16), though a recent study in Nigeria found that the prevalence of *Histoplasma* antigenuria (indicating probable disseminated disease) was 7.7% among people with advanced HIV disease and a clinical syndrome compatible with histoplasmosis (14). Histoplasmin skin test prevalence surveys have indicated exposure ranging from 0.0% to 35% in different African countries (reviewed in reference 14). Endemicity of histoplasmosis across a variety of African climatic zones (17, 18) suggests the possibility of extensive genetic variation (16).

Based on limited case series, Africa was hypothesized to harbor three lineages of *Histoplasma* at the taxonomic rank of variety (below species), which in turn have been hypothesized to cause three disease forms. *Histoplasma capsulatum* var. *duboisii* (*Hcd*) was ascribed as the causal agent of African histoplasmosis in humans, with predominant skin, lymph node, and bone involvement, and most case reports from central and western Africa (19–22). *Histoplasma capsulatum* var. *farciminosum* (*Hcf*) was considered the causal agent of epizootic lymphangitis characterized by ulcerated lesions of the skin and pyogranulomatous subcutaneous nodules in equines. This occurs in Africa, Asia, the Middle East, and Mediterranean regions. Finally, *H. capsulatum* var. *capsulatum* (*Hcc*) included New World human pathogens that cause the classical form of pulmonary histoplasmosis and was hypothesized to be found worldwide, especially in Latin America and in the Midwest of the USA.

Our understanding of the extent of genetic variation within *Histoplasma* has undergone extensive revision. The use of phylogenetics with multilocus sequence typing (MLST) revealed that this classification, based largely on phenotypic characteristics, was inadequate and that *Histoplasma* might harbor several previously unrecognized phylogenetic species (1, 23–26). Of note, these MLST studies indicated that *Hcc* harbored at least eight phylogenetic species but suggested the existence of an African lineage of *Histoplasma* consistent with *Hcd*. On the other hand, *Hcf* appeared as a non-monophyletic group, as animal-derived samples appeared to be distributed in three different phylogenetic species (1). Since then, the *Hcf* group has been considered artificial. Genetic sampling of these two lineages (i.e., *Hcd* and *Hcf*) has been sparse since then in terms of isolates and genetic markers.

Whole-genome sequencing data combined with population genomic approaches confirmed that at least four *Histoplasma* clades are genetically isolated and sufficiently diverged to be considered phylogenetic species. These results led to the proposal of a taxonomic reorganization of the genus: (*i*) *Histoplasma capsulatum sensu stricto* (*ss*) (27), (*ii*) *Histoplasma mississippiense* sp. nov., (*iii*) *Histoplasma ohiense* sp. nov., and (*iv*) *Histoplasma suramericanum* sp. nov. (28). Systematic characterization revealed that these species differ both genomically and phenotypically (29). Further studies revealed the existence of two additional phylogenetic species: one endemic to Southern Brazil (30) and another one endemic to the Indian subcontinent (31). One blind spot in most efforts to characterize the genetic diversity of *Histoplasma* has been the paucity, or even absence, of African samples (13, 15, 18). To date, all phylogenomic assessments have used just a handful of African isolates (28, 31). The neglected nature of histoplasmosis in Africa, due to lack of accurate and timely diagnostic methods (17, 18, 32, 33), has historically compounded this issue.

In this study, we address this gap using a population genomics approach to characterize isolates from a South African collection and a set of isolates diagnosed in Europe from individuals with a history of spending time in Africa. We found that African strains belong to one of three groups. The largest cluster is of human clinical origin, often showing skin involvement, a manifestation compatible with African histoplasmosis. The group is genetically similar to the phylogenetic species previously described as the *Africa* lineage (28). Given the prevalence of skin involvement, a classical manifestation of African histoplasmosis (15, 16), we believe this group corresponds to the classically defined *Hcd*. A second small group (*Hcf*) is responsible for cases of epizootic lymphangitis in equines. These two lineages are differentiated enough to be considered phylogenetic species and are not closely related to each other. A single African isolate appears closely related to a phylogenetic species, mz5-like, previously described in South America (34). We use these genome-wide data to study patterns of selection and introgression in these newly identified lineages and find instances of parallel positive selection; we also reveal that the *Africa* lineage has undergone a population size increase since speciation. Next, we compared patient characteristics of histoplasmosis caused by the *Africa* lineage with those of other *Histoplasma* spp. and found that the disease was more frequently associated with extrapulmonary manifestations than other lineages of *Histoplasma*, but cases did not differ in terms of the median age, patient sex, or the proportion of patients living with HIV. Finally, we used climatic projections to assess the potential range and range expansion scenarios of the disease. Our results suggest the need to implement surveillance to monitor the different phylogenetic species of *Histoplasma* across Africa.

## MATERIALS AND METHODS

### South African isolate collection

The samples included in this study came from two sources. First, we recovered isolates from the collection of *Histoplasma* spp. isolates archived between 1975 and 1998 at the National Institute for Communicable Diseases (NICD) in South Africa. A total of 36 isolates were stored in water at room temperature over these years; only 3 isolates were viable at the time of study. The second source of isolates was a passive laboratory-based surveillance for thermally dimorphic fungi associated with human disease conducted by NICD from 2010 to 2021 at South African diagnostic pathology laboratories in the public and private sectors. A case of histoplasmosis was defined as a patient with an isolate, cultured from any specimen type, which was confirmed as *Histoplasma* spp. by phenotypic or molecular methods. During this period, 13 human and two equine clinical isolates were collected, described phenotypically, and sequenced for further genomic analysis (see below). An additional sample, T-3-1, was isolated in Texarkana, USA (ATCC 22635 [35]), and was donated to the South African collection as a mating-type tester strain. *Histoplasma* isolates were stocked and maintained at the NICD from 2010 to 2021. Once isolates were stable, we stored them as slants at room temperature and glycerol stocks at −70°C.

### Cultures

We subcultured all archived samples directly onto Sabouraud dextrose agar containing cycloheximide/Actidione (Diagnostic Media Products [DMP], Sandringham, South Africa) and incubated at 25°C for up to 6 weeks for the mold phase. To confirm dimorphism, all isolates were transferred to brain heart infusion (BHI) agar (DMP) and incubated at 35°C for up to 4 weeks for conversion to the yeast phase. All cultured isolates were handled in a biosafety level 3 (BSL3) laboratory. Yeast and mold phase isolates were examined using both light microscopy and electron microscopy (EM) (see below).

### Microscopy

To study the microscopic features, we obtained culture aliquots from the culture conditions described above. For both yeast and mold cultures, we sampled the culture after 2 to 6 weeks of growth, respectively, and the samples were visualized using a light microscope with lactophenol blue stain in BSL2 and BSL3, respectively. The samples were observed in an Olympus BX53 light microscope with ×1,000 magnification.

To visualize yeast and mycelial samples with EM, we followed previously described protocols (36). Briefly, we fixed samples from six isolates in 2.5% glutaraldehyde in 0.2 M phosphate buffer at pH 7.1 for at least 2 hours. We then followed a standard processing protocol which involved the following steps: pieces of agar containing the fungi were excised and rinsed in buffer, then post-fixed for 2 hours in 1% osmium tetroxide, after which the samples were dehydrated in a series of ethanol solutions. For transmission EM, the specimens were infiltrated with a low viscosity epoxy resin, polymerized, and sectioned to 70 nm thickness using a Leica EM UC6 ultracut microtome. They were then stained with uranyl acetate and lead citrate. The dehydrated specimens were treated with hexamethyldisilazane, air-dried overnight, mounted on stubs, and coated with carbon. The specimens were analyzed using a Zeiss Crossbeam 540 scanning electron microscope at 1 kV, with an Olympus Quemesa CCD camera and OSIS calibrated software at 80 kV.

### Genome sequencing

#### DNA extraction

We extracted the DNA from the 18 human/ equine clinical isolates described above from NICD. We grew isolates in their yeast form (35°C) on BHI agar for 4 weeks. We collected 1 g of culture and extracted high-molecular-weight genomic DNA using the Zymo ZR Fungal/Bacterial DNA Miniprep Kit (Zymo Research Corp., USA) following manufacturer’s instructions. We visualized the integrity of the extracted DNA with agarose gels. Additionally, we cultured and extracted DNA samples from isolates collected at the Institut Pasteur in France derived from patients of African origin diagnosed in France (*n* = 10). Finally, we followed a similar approach to obtain DNA from the Spanish collection of African *Histoplasma* (*n* = 8) described in reference 16. The protocols for these extractions were similar to the one described above, but the protocols were carried out at the Institut Pasteur and in the Centro Nacional de Microbiología, Instituto de Salud Carlos III, Madrid, Spain, respectively. The details of the patients diagnosed in Europe are listed in Table S1.

#### Species identification with PCR

Before submitting isolates for whole-genome sequencing, we used Sanger sequencing to verify if the isolates indeed belonged to the genus *Histoplasma*. We amplified the *ITS* locus using primers and PCR as previously described (37). We then performed Sanger sequencing on the resulting amplicons (forward and reverse) to confirm the identity of the isolates as *Histoplasma*. The amplicons were sequenced using a 3130 sequencer (Applied Biosystems, Life Technologies Corporation, USA). We visually inspected the chromatograms using Chromas software (https://technelysium.com.au/wp/chromas/). We used the Basic Local Alignment Search Tool in GenBank (https://www.ncbi.nlm.nih.gov/) to find the most similar sequences to each individual sequence. The ITS region of all 36 isolates (i.e. 18 from South Africa, 10 from France, 8 from Spain) indicated that all isolates belonged to the *Histoplasma* genus.

#### Library construction

DNA samples from 28 isolates (18 from the NICD collection and 10 from Institut Pasteur) were processed for genomic analysis in South Africa at the NICD. After DNA extraction, paired-end genomic libraries were built from high-molecular-weight DNA following the protocol described by Illumina’s MiSeq System Denature and Dilute Libraries Guide. Approximately 10 µg of DNA was sonicated with a NextSeq DNA library kit to a mean fragment size of 150 bp. We evaluated the concentration of each library with a Kapa library quantification kit (Kapa Biosystems, Wilmington, MA, USA) on a 7900HT Instrument (Life Technologies). The eight samples from the Spanish collection (in reference 16) were prepared in the same way at the sequencing center at Northern Arizona University.

#### Sequencing

We sequenced the libraries from the South African and French collections using v3 or v4 chemistries (read length = 100 bp) on an Illumina NextSeq instrument at the NICD sequencing core facility in Sandringham, Johannesburg. We obtained paired-end reads for all isolates and targeted an approximate coverage of 100×. The quality of each read was determined using the NextSeq Control software version 2.0.5 in combination with FASTQC and MultiQC Software (38, 39). The target average coverage for each read was 100×. To sequence the samples from reference 16, we used an Illumina MiSeq platform at Northern Arizona University. We used FASTQC version 0.11.9 (38) to check initial read quality and Trimmomatic 0.39 (40) to remove adapters and regions of low quality at the read ends. We followed an iterative approach, and after a first round of trimming, we checked individual read quality using FASTQC. We sequenced DNA from all the 36 isolates of the collection described above. Two genome sequences (SA4 and SA1356) were excluded from any downstream analyses as they had lower coverage than any of the other samples (<20×). Table S1 lists geographical details, sequencing coverage, and accession numbers for each of the 34 newly sequenced genomes (and the two sequenced isolates with low mapping quality).

### Publicly available data sets

We used Illumina reads from previous studies to contextualize the genetic variation in the samples from Africa. We used two samples from Africa (28) and 78 sequences of non-African *Histoplasma* isolates. The non-African isolates belonged to five different lineages: 10 from *H*. *mississippiense*, 11 from *H*. *ohiense*, 16 from the *Histoplasma* lineage endemic to India, 11 from *H*. *suramericanum* (34), 8 from *Amazon-I*, 1 from *mz5-like* (34), and 21 from *H. capsulatum ss* (28, 30, 31). Table S2 lists all the Sequence Read Archive (SRA) accession numbers for the previously published *Histoplasma* sequences used in this study. To root the phylogenetic trees of *Histoplasma*, we used 13 genomes from the family Ajellomycetaceae as outgroups: 2 genomes from the species *Emergomyces* (formerly *Emmonsia*) *crescens* (41), 5 from the genus *Blastomyces* (41–43), and 6 genomes from the genus *Paracoccidioides* (44). Table S3 lists all the SRA accession numbers for the previously published sequences used in this study.

### Mapping, cleaning, filtering, and variant calling

We followed the same approaches described in reference 31 to map reads and identify single-nucleotide polymorphism (SNP) variants. Briefly, FASTQ files were mapped to reference *H. mississippiense* WU24 (45) using BWA version 0.7.15 (46). The resulting BAM files were cleaned and sorted, and duplicate reads were marked using Samtools version 1.11 (47). SNPs were called using the GATK version 4.1.7.0 HaplotypeCaller function, setting the -ploidy option as 1 (48). The resulting g.vcf files were merged using the GATK GenomicsDBImport function and subsequently joint genotyped with the GATK GenotypeGVCFs function, setting the -ploidy option as 1 as well. We used bcftools (49) to only include biallelic SNPs. Finally, we applied the following filters to the resulting multisample VCF files using the GATK VariantFiltration function: quality depth <2.0, FisherStrand >60.0, mapping quality <30.0, mapping quality rank sum <−12.5, and read position rank sum <−8.0. We generated one multisample VCF for all sequences.

### Phylogenetic reconstructions

We used phylogenetic analysis to determine whether the genetic clusters found in African *Histoplasma* satisfied the requirements to be considered phylogenetic species. The previously described phylogenetic species concept was followed, defining species as genetic clusters that are reciprocally monophyletic and for which there was genealogical concordance across genome-wide unlinked loci (50, 51).

First, we generated a concatenated whole-genome maximum likelihood (ML) tree. Briefly, we used the Python script *vcf2phylip* (52) to convert each multisample VCF into a concatenated genome-wide alignment. We then built ML trees from the genome-wide concatenated alignment using IQ-TREE version 2 (53, 54). We used GTR-gamma as the substitution model in all trees. To assess branch support, we used 1,000 replicates of ultrafast bootstrap (55).

Second, we measured the extent of genealogical concordance throughout the genomes in each population. We split the supercontigs into 100 kb genetic windows. We converted the genomic window sequences into phylip format using the Python script *vcfphylip* (52); we then used IQ-TREE version 2 (53, 54) to generate a ML tree for each genomic window. These genealogies were then used to calculate concordance factors (CFs) (56, 57), which reflect the proportion of genealogies that are consistent with the species tree. Species at advanced stages of divergence usually show concordant differentiation across the whole genome and not just in a few loci (51). We also used these window-based genealogies to calculate the quadripartition support for each quartet using ASTRAL (58, 59). We followed a similar approach to calculate CF and quadripartition support using BUSCO genes instead of genomic windows. We identified each conserved orthologous gene in the database Onygenales Odb10 BUSCO (60, 61) with 4,183 Ajellomycetaceae genes and extracted the sequences of these genes from the multisample VCF. All other analyses were the same as described for genomic windows. These analyses are aimed at determining whether different genomic regions along the genome show the same evolutionary history.

### Genetic differentiation and gene flow analysis

We also used population genetics metrics to quantify the level of differentiation of the *Histoplasma* lineages present in Africa. First, we used principal component analysis (PCA) to measure allele frequencies across potential lineages of *Histoplasma*. This method summarizes large high-dimensional data sets with possibly correlated variables into a reduced set of independent variables, enabling easy visualization. The principal components (PCs) of all the samples are then projected on a two-dimensional space, and the axes show the different variables. Clustering is based on correlations between closely related individuals revealing population relations and within-population stratification. We used, cleaned, and sorted BAM files with the program *angsd* version 0.921 (62) to generate a *beagle* file with estimated genotype likelihoods. We filtered sites with more than 20% missing data, a mapping quality of <30, and a base quality of <20. Then we used the program PCAngsd (63) to compute the covariance matrix among sites and visualize the principal components. We plotted the combinations of PC1/PC2 and PC3/PC4. We evaluated the contribution of each PC using a Scree plot in which the contribution of each eigenvalue was drawn along the percentages of variance explained following a broken stick model (i.e., a benchmark of the variance expectations if the components were randomly distributed) and a uniform distribution model.

Second, we used vcftools (64) to generate a VCF with variant and invariant sites (gvcf, --max-maf 0). Using this gvcf file as an input, we used *Pixy* (65) to calculate heterozygosity (*π*) within lineages and Dxy between the monophyletic lineages identified in the phylogenetic tree (see above); we also calculated the extent of genetic divergence and diversity along the genome in 1,000 bp windows. To compare the values of *π* in each of these lineages with the magnitude of pairwise Dxy_xy_, we used an approximative two-sample Fisher-Pitman Permutation test (R function oneway_test, library “coin” [66, 67]) with 1,000 subsampling iterations.

Finally, we tested the magnitude of gene exchange between the putative phylogenetic species from Africa and other *Histoplasma* lineages. Species that are at an advanced stage along the divergence process show none or low amounts of gene flow between species. We estimated the extent of admixture by calculating the metric f_d_ published in reference 68. Previous simulations have shown that f_d_ is a powerful metric to detect gene flow between populations. We plotted the statistical values in R version 4.3.0 (69) for visualization. Finally, we calculated the *f*_b_ heuristic (70), which summarizes the *f*_4_-admixture ratios calculated by *Dtrios* to identify and plot introgression events between specific nodes, or tips, in a phylogenetically informed manner. We plotted the results from this analysis with f-branch, one of the programs included in *Dsuite* (71). *f*_b_ is not a percentage of introgression and instead shows the support for introgression events in multispecies data sets (70). We used the ML whole-genome tree described above as an input, but we obtained similar results when we used the ASTRAL tree.

### Selection along the genome

Finally, we studied the influence of selection on allele evolution in genomes of the two main African lineages of *Histoplasma* (referred to as *Africa* [corresponding to *Hcd*] and *Hcf* respectively). We measured lineage-specific differentiation with the population branch excess (PBE) (72) to scan for signatures of selection. Instances of selection at a given locus will show significantly longer branches for the lineage that has experienced selection. Leveraging information from our whole-genome ML tree, we estimated PBE, a metric of positive selection, with the program *PBScan* (73) using the *Africa-H. ohiensis-Histoplasma capsulatum ss* and *Hcf-H. ohiensis-H. capsulatum* trios. The first one is tailored to identify genes under positive selection in the *Africa* branch; the latter is tailored to identify selected genes in the *Hcf* branch (see Results). *PBScan* analyzed non-overlapping windows containing 100 SNPs (15 kb long on average) from the *beagle* file produced with ANGSD to estimate absolute levels of differentiation (Dxy) and transform them into relative divergence times (*T*) in each population pair. We used the relative divergence times to calculate the PBE (74), a modified version of the population branch statistic (PBS), to detect the branch outliers in the *Africa* and *Hcf* lineages, compared to their predicted value.

To assess whether these genomic windows show an enrichment for any type of gene category, we did Gene Ontology (GO) analyses using ShinyGO version 0.77 (75) and compared against the GO biological databases with a 0.05 false discovery rate (FDR), a pathway minimal size of 2, and a maximum of 2,000. To minimize the influence of taxon-specific annotations, we redid the analyses against the databases of four different species: *Saccharomyces cerevisiae*, *Kluyveromyces lactis*, *Schizosaccharomyces pombe*, and *Nakaseomyces glabratus*.

### Demography

Next, we studied the population size of the *Africa* lineage of *Histoplasma* since its split from a sister phylogenetic species, *H. capsulatum sensu stricto*. First, we studied whether the *Africa* lineage showed population structure, and this phenomenon can be misleading when inferring divergence dates. We used a PCA as described above (genetic differentiation and gene flow analysis) (76). We used GADMA (77), which uses a genetic algorithm to infer the demographic history from the joint allele frequency spectrum. Since we detected three populations within the *Africa (Hcd)* lineage (see Results), we ran three different analyses. GADMA was run with two populations: *H. capsulatum ss* (21 samples) and the *Africa* lineage (between 2 and 22 samples, depending on the population). Since not all samples had genotype data for every site, the allele frequency spectrums were projected using EasySFS (https://github.com/isaacovercast/easySFS [78]). We ran EasySFS on the input vcf file to generate a preview of the number of segregating sites for each projection level. We then identified the projection levels that maximized the number of segregating sites for each of the two lineages. EasySFS was then run with these projections to generate a joint site frequency spectrum file. The resulting data set included 17 and 18 samples for *H. capsulatum ss* and the *Africa* lineage, respectively. GADMA was run with this file, default parameters, and a genomic sequence length of 37,996,987 bp corresponding to the length of the *H. mississippiense* WU24 reference genome (45). We also assumed a mutation rate of 5 × 10–10 bp/generation, which is the range of the parameter when measured in mutation accumulation lines in other ascomycetes (79, 80).

### Comparison of clinical presentation of histoplasmosis caused by different *Histoplasma* lineages

We studied whether histoplasmosis presentation caused by the *Africa* lineage was comparable to that of the disease caused by other phylogenetic species of *Histoplasma*. The patients we described in this study and that we used for these analyses are listed in Table 1. Table S4 lists the studies that have reported information for other *Histoplasma* lineages. We did four analyses using this data set. First, we compared if the *Africa* lineage had a similar proportional representation of skin lesions vs pulmonary lesions compared to other species. We looked at whether African histoplasmosis was associated with skin involvement more often than with other *Histoplasma* phylogenetic species; skin involvement is considered a classical manifestation of African histoplasmosis even in the absence of immunosuppression (13). We used a two-sample test for equality of proportions with continuity correction (prop.test function, R package “stats” [81]). We focused on pairwise comparisons involving the *Africa* lineage and did five pairwise comparisons; we adjusted the *P* values accordingly using a Holm correction (function p.adjust, R package stats [81]). We calculated the power of the proportion tests using the function pwr.2p2n.test (R package “pwr” [82]). We restricted all pairwise comparisons to phylogenetic species that had at least nine observations (other species had data for fewer than five patients). We used a similar approach to test whether the sex ratio of histoplasmosis patients was equal across different lineages of *Histoplasma* and whether the proportion of patients who were living with HIV differed among *Histoplasma* phylogenetic species.

**TABLE 1:** Characteristics of South African human patients (*n* = 13) and horses (*n* = 2) with confirmed histoplasmosis from this study (2010–2021), and human/ equine cases from other published studies (*n* = 5)^*a*^

Source	Year of isolation	Isolate ID	Sex	Age (years)	HIV serostatus	Specimen type	Reference
Human (*n* = 17)	2010	SA46	Female	17	Unknown	Skin punch biopsy	This study
	2010	SA47	Male	30	Unknown	Skin punch biopsy	This study
	2013	SA297	Female	27	+	Skin punch biopsy	This study
	2013	SA302	Female	28	+	Tissue	This study
	2014	SA355	Male	46	+	Skin	This study
	2014	SA562	Male	41	+	Skin punch biopsy	This study
	2015	SA602	Male	40	+	Tissue	This study
	2015	SA811	Male	39	+	Tissue	This study
	2018	SA1356	Male	42	+	Skin	This study
	2019	SA1371	Female	37	+	Skin	This study
	2019	SA1436	Female	43	+	Blood	This study
	2020	SA1556	Female	39	+	Tissue	This study
	2021	SA1704	Female	38	+	Skin punch biopsy	This study
	2012	CM7001	Female	32	+	Serum	(16)
	2015	CM 7704	Male	35	+	BAS	(16)
	2015	CM7767	Male	50	+	Adenopathy	(16)
	2016	CM8013	Male	47	+	Skin biopsy	(16)
Horse (*n* = 3)	2019	SA19VMG	–	–	–	–	This study
	2020	SA20VMK	–	–	–	–	This study
	–	CBS 536.84	–	–	–	–	(16)

^*a*^ Sex, age, and HIV serostatus are reported only for human patients; + indicates HIV seropositive patient. – indicates data not available.

Finally, we compared the age of the histoplasmosis patients affected by each of the six *Histoplasma* spp. We used one-way type III analysis of variance (ANOVA) (function Anova, R package “car” [83]) with age as the response variable and *Histoplasma* spp. as the single fixed factor. We followed the ANOVA with Tukey’s honestly significant difference post hoc pairwise comparisons (function glht, library “multcomp” [84, 85]) to identify whether any lineage differed from the others.

### Bioclimatic niche modeling

We leveraged data from the WorldClim database (86, 87) to model the environmental conditions associated with *Histoplasma* in Africa. We estimated the range of climate conditions associated with the reported cases of histoplasmosis in Africa by randomly sampling 1,000 localities within each of the 31 African countries for which we have caseload data (from references 14 and 88). We used WorldClim data, with a 2.5 arcminute resolution, to describe the breadth of climatic conditions within each of the countries from which histoplasmosis has been reported. We extracted the mean annual temperature (Bio1), diurnal temperature range (Bio2), maximum temperature of the warmest month (Bio5), minimum temperature of the coldest month (Bio6), and mean annual precipitation (Bio12) for each of the sampled locations using the “extract” function in the “raster” package in R (89). We used the “scale”’ function to center and scale each of these niche axes by subtracting each variable mean and dividing by its standard deviation. We then calculated five-dimensional niche hypervolumes as a multidimensional measure of climatic niche breadth (following Hutchinson’s definition of the niche and an *n*-dimensional space [90]). We used the “hypervolume” function in the “hypervolume” package (91) with each scaled variable as a niche axis. Briefly, this method uses Gaussian kernel density estimation in *n-*dimensions to describe the bounds, shape, and volume of a point cloud in high-dimensional space (e.g., in multidimensional climate space). In our case, we treat these hypervolumes as a measure of climatic niche breadth in the five-dimensional climate space that we analyzed. We used a generalized linear model to ask whether niche hypervolume is a function of land area using the “lm” function in the stats package (81). Finally, we used another series of generalized linear models to test whether niche hypervolume or the per-country mean values of any of the individual climate axes (Bio1, Bio2, Bio5, Bio6, or Bio12) predict histoplasmosis caseload (lm function in the stats package). We also tested whether the breadth of any of the climate axes (calculated as the middle 95% quantile range) is predictive of caseload with a set of generalized linear models in which caseload was the response variable and each bioclimatic niche variable (Bio1, Bio2, Bio5, Bio6, or Bio12) was the predictor (lm function in the stats package [81]).

Next, we used projections of future climate conditions to construct predictive models for the future habitat suitability for pathogenic *Histoplasma*. We used the MIROC6 Global Climate (92, 93) Model downloaded from the WorldClim database (86), projected across three models of anthropogenic warming (Shared Socio-economic Pathways [SSPs]; SSPs 126, 370, and 585) from the WorldClim database. These models encompass three outlooks on future climatic warming (modest, moderate, and extreme, respectively), induced by increasing amounts of atmospheric CO_2_. We lacked the kind of specific locality information that is required for a more traditional maximum entropy (MaxENT) approach (e.g., see references 94 and 95) for each of the cases in our study, so we developed a novel approach to quantify climatic suitability of histoplasmosis infections. To approximate the most suitable climatic range for histoplasmosis infections, we constructed a MaxENT (96, 97) model based on 1,000 randomly sampled geographical points within each of the three African countries with the greatest caseloads (Nigeria, the Democratic Republic of the Congo, and South Africa). We generated the random points using the “st_sample” function in the “sf” package (94), followed by the “st_coordinates” function to extract latitude and longitude for each point, and we used these samples as pseudo-observations to construct the MaxENT model. In essence, our approach uses the climatic breadth within these predictor countries as a proxy for the climatic requirements of the pathogen because current sampling efforts prevent us from assessing the climatic breadth of diagnosed cases directly. The model is based on bioclim variables from current climate conditions, providing a prediction of climate suitability across the African continent similar to 98. Future suitability projections were carried out by applying the MaxENT model to climate rasters representing three future time intervals, 2021–2040, 2041–2060, and 2061–2080. The MaxENT model was implemented using the “maxent” command in the “dismo” package in R (99) and applied to the broader region (Africa, Southern Europe, and the Middle East) at 2.5 arcminute resolution to predict how histoplasmosis infections might shift across the landscape in response to warming. To quantify the proportion of the study region that is suitable for histoplasmosis, we considered suitability values from MaxENT <0.4 to be low suitability, between 0.4 and 0.6 to be moderate suitability, and >0.6 to be highly suitable following (100). We measured the proportion of land area that fell into each of those categories, relative to the total land area in the study region.

## RESULTS

### Africa harbors at least three lineages of *Histoplasma*

**Fig 1:**
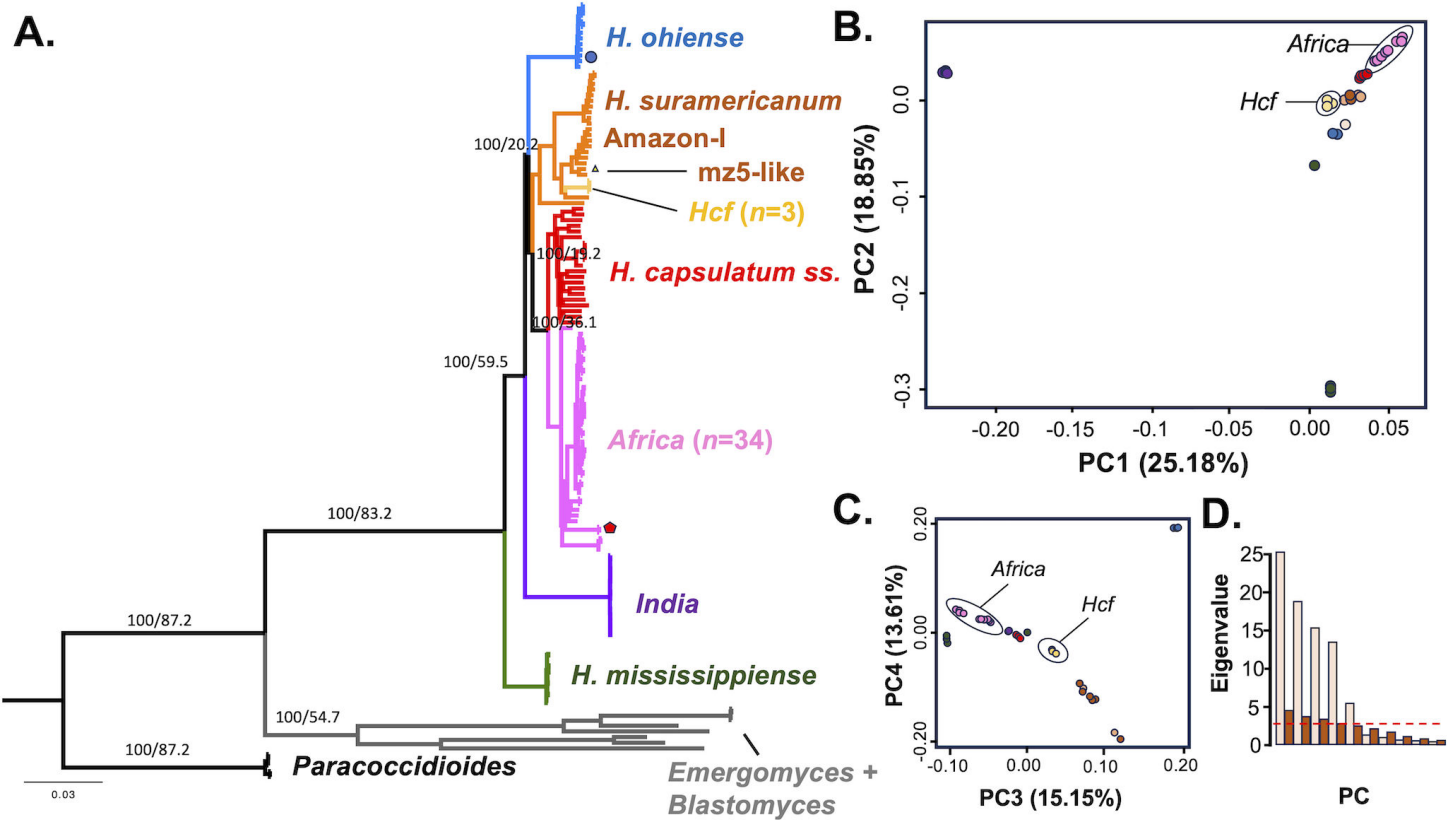
The partition of genetic diversity in *Histoplasma* shows that the *Africa* and *Hcf* lineages are differentiated monophyletic groups. (**A**) BUSCO tree showing the genealogical relationships between the different lineages of *Histoplasma*. Isolates collected in Africa belong to two groups, *Africa* and *Hcf*, both marked with black bars. T-3-1, a North American isolate, is marked with a blue circle. All the samples in the *Africa* lineage are newly sequenced with the exception of the two tips labeled with a red pentagon (Hc_duboisii-A and Hc_duboisii-B). The only isolate from Africa that clustered outside of the *Africa* and *Hcf* lineages clustered with mz5-like (a species described in reference 34) and is labeled with a yellow triangle. Numbers in the nodes show the bootstrap and concordance factors for each node. (**B**) The first two components (PCs) of a principal component analysis show differentiation of the *African* lineages with the rest of genetic clusters in *Histoplasma* and explain over 40% of the genetic variance. (**C**) PC3 and PC4 explain 29% of the variance. (**D**) Scree plot showing the contributions of each PC. Light tan bars show the value of the eigenvalues. Brown bars show the expected eigenvalues in a broken stick model, and the red dashed line shows the expected contributions of the eigenvalues in a uniform distribution model.

We explored the genetic diversity of *Histoplasma* on the African continent using phylogenomic approaches. In this study, we include 36 isolates of African origin and their genomes and 96 non-African isolates. First, we generated a maximum likelihood phylogenetic tree to study the phylogenetic relationships between African *Histoplasma* and isolates from the rest of the world using whole-genome sequences (Fig. 1A). As expected, T-3-1—an isolate in the South African collection but originally collected in the USA—clustered with *H. ohiense*. African *Histoplasma* isolates do not form a single monophyletic group and instead belong to three different groups. One isolate belongs to the mz5-like lineage initially reported only from South America (34). The isolate was collected in Africa from a 63-year-old male patient who had undergone a solid organ transplant ([16] isolate CM-5788). The mz5-like clade showed a high level of support, even with the inclusion of this isolate (bootstrap support = 100%, CF = 77.5). No further travel details exist for this patient. All the other African isolates form two monophyletic groups not closely related to each other. The larger group (*n* = 34) represents the *Africa* spp. described in Sepúlveda et al. (28), which seems to be equivalent to the lineage previously known as *H. capsulatum* var. *dubosii* (sometimes referred to simply as *H. duboisii*). This clade is highly supported both by bootstrap (100%; 1,000 ultrafast replicates) and CFs based on BUSCO gene genealogies (77.2%, Fig. 1A). The *Africa* lineage is closely related to *H. capsulatum sensu stricto*, which is consistent with previous studies (Fig. 1A). A second smaller group of African strains (*n* = 3) was also monophyletic (Fig. 1) and highly supported (100%, 1,000 ultrafast replicates; CF = 72.2%). Notably, this clade encompassed the three veterinary clinical strains isolated from horses with equine lymphangitis (SA19VMG, SA20VMK, and CBS 536.84—also known as CM-7436—the neotype of *H. capsulatum farciminosum*). The two clades appear well differentiated in the first four PCs of the PCA (Fig. 1B and C), which explained the majority of the genetic variance (Fig. 1D). Herein, we use the nomenclature for these two lineages when originally proposed and denominate the African histoplasmosis lineage: *Africa* (as in reference 28), and the equine histoplasmosis *Hcf*. An ASTRAL tree which compiles the signal from gene genealogies generated from genomic windows also showed the two lineages have a high quadripartition support (1.0), which suggests that a large portion of the genome shows the two groups as monophyletic groups (Fig. S1). BUSCO-based concordance analyses show similar results to those obtained based on genome windows (Fig. S2). This level of concordance is expected in cases in which there is advanced divergence and thus comparatively little admixture or incomplete lineage sorting (51). Topologies using a different reference genome (Fig. S3) vary in the reciprocal relationships between species, but in all cases, the *Africa* and *Hcf* lineages form monophyletic groups; *Africa* is always sister to *H. capsulatum ss*; and *Hcf* always appears as a monophyletic group nested within a cluster of previously reported South American phylogenetic species (34, 101).

**Fig 2:**
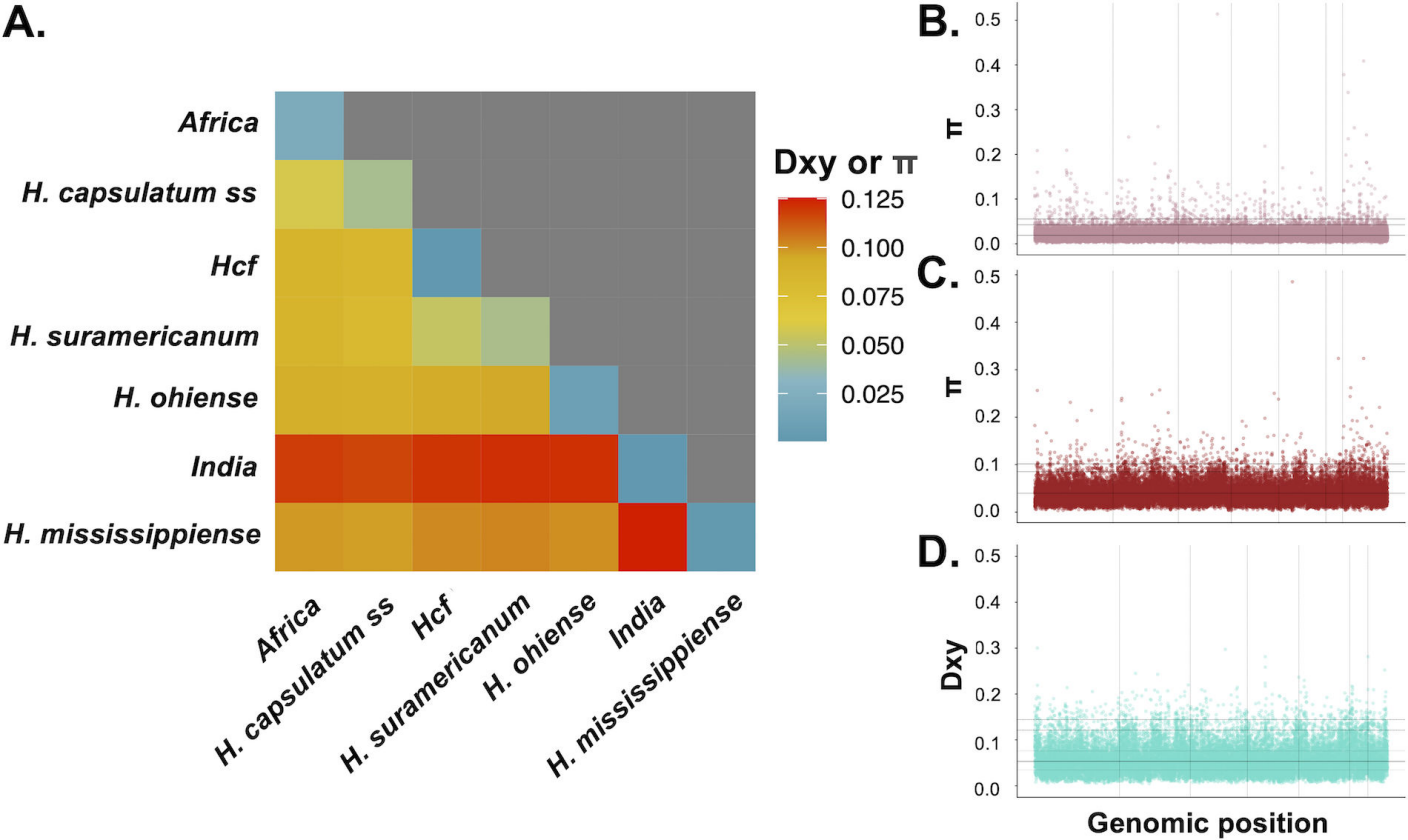
Genetic variation and differentiation between *Histoplasma* spp. (**A**) Nucleotide diversity analysis of seven *Histoplasma* spp. In all cases, Dxy is larger than pairwise *π*. (**B**) *π* along the genome in *Hcd*. (**C**) *π* along the genome in *H. capsulatum ss*. (**D**) Dxy between *Africa* and *H. capsulatum sensu stricto* shows extensive genome-wide differentiation between the two clades. Figure S4 shows *π* along the genome in *Hcf*, and Fig. S5 shows Dxy between *Africa* and *Hcf*.

Population-genetics-based approaches also revealed that the two better-sampled lineages we find in Africa (i.e. *Africa* and *Hcf*) are genetically distinct from other lineages and from each other. We quantified the extent of genetic divergence (Dxy) between all pairs of *Histoplasma* lineages. Both *Africa* and *Hcf* show a high degree of differentiation from all other lineages (Fig. 2A). We calculated heterozygosity (*π*) within each lineage, which measures how differentiated individuals are within a clade. Unlike the only previous studies that sampled genomes of a limited number of isolates and inferred low polymorphism (*N* = 3, *π* = 3.290 × 10^–6^ [28]), we find the existence of extensive genetic variation within the group (*π* = 0.021, CI = 1.837^−4^; Fig. 2A). The *Hcf* lineage shows a much lower level of genetic variability (*π* = 9.390 × 10^−4^, CI = 1.766 × 10^−4^; Fig. 2A), but note the sample size is low (*N* = 3). We compared the magnitude of inter-lineage divergence with the magnitude of genetic variance within lineages. Instances of advanced speciation show larger Dxy values than *π* (51, 102, 103). Indeed, both the better-samples African lineages show a much larger Dxy when compared to all the other *Histoplasma* lineages than the *π* value of any lineage (approximative two-sample Fisher-Pitman permutation test, *P* < 0.05; Fig. 2A). Both *π* and Dxy were evenly distributed along the genome in both lineages (Fig. 2B through D; Fig. S4 and S5). These metrics of genetic differentiation and diversity indicate that the *Africa* and *Hcf* lineages not only are monophyletic clades but also show advanced differentiation from other lineages of *Histoplasma*.

**Fig 3:**
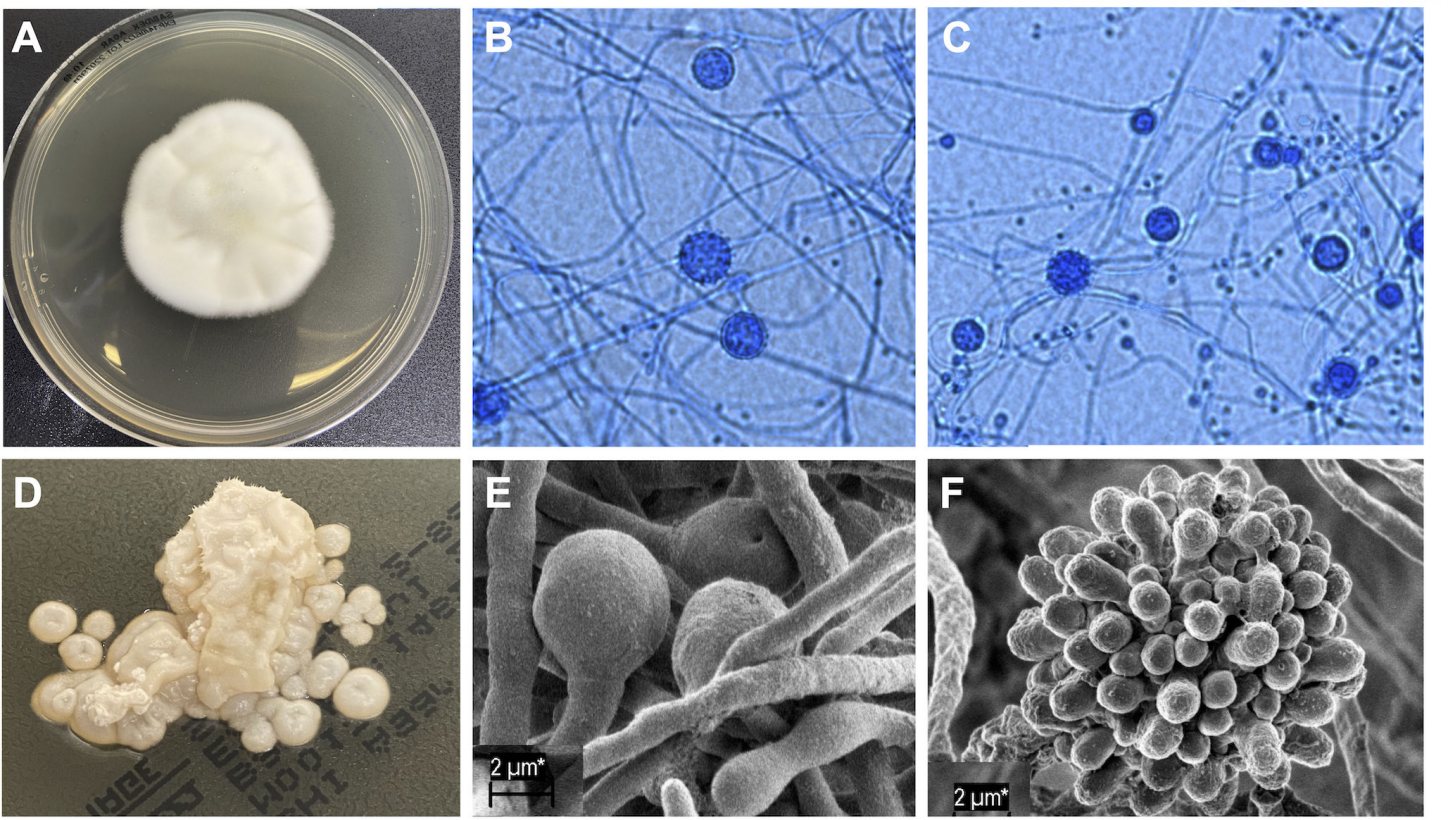
Morphological characteristics of the *Histoplasma Africa* samples. We show the morphology of isolate SA1704, but all other isolates show similar characteristics. (**A**) Morphology of a 3-week-old *Africa* isolate growing on a plate of Sabouraud dextrose agar at 25°C. (**B**) Light microscope slide stained with lactophenol blue showing the mycelial stage of *Histoplasma Africa*. (**C**) A similarly prepared slide for T-3-1 which belongs to *H. ohiense*. (**D**) Culture of a 2-week-old *Africa* growing on brain heart infusion agar at 35°C. (E) Slide of electron microscopy image of a 2-week-old *Africa* (SA0297) growing on Sabouraud dextrose agar at 25°C showing the microconidia of mold form (magnification = ×2,000, extra high tension = 5.00 kV, working distance = 9.2 mm, Signal A = SE2, bar 10 µm). (F) Electron microscopy image of a 2-week-old *Hcf* (SA20VMK) growing on Sabouraud dextrose agar at 25°C showing a tuberculate macroconidium (magnification = x2,000, EHT = 5.00 kV, WD = 9.2 mm, Signal A = SE2, bar 10 µm).

We also examined the general culture and microscopic appearance of the two lineages. The 16 *Africa* lineage isolates and the two *Hcf* lineage isolates from South Africa show a similar colony morphology. At 25°C, colonies were white, floccose, and mold-like, which is consistent with the classical culture and microscopic morphology of *Histoplasma* (Fig. 3). When cultures were grown at 37°C, the fungus transitioned into cream to brown colonies (Fig. 3D). Figure 3E and F show electronic microscopy of the mold phase, which revealed hyphae with round microconidia and spiked macroconidia. As expected, yeast colonies (Fig. 3D) were composed of oval budding yeast cells (Fig. S6D and E). Figure S6 shows the *Hcf* colony morphology.

### The *Africa* lineage has undergone a population expansion over time

**Fig 4:**
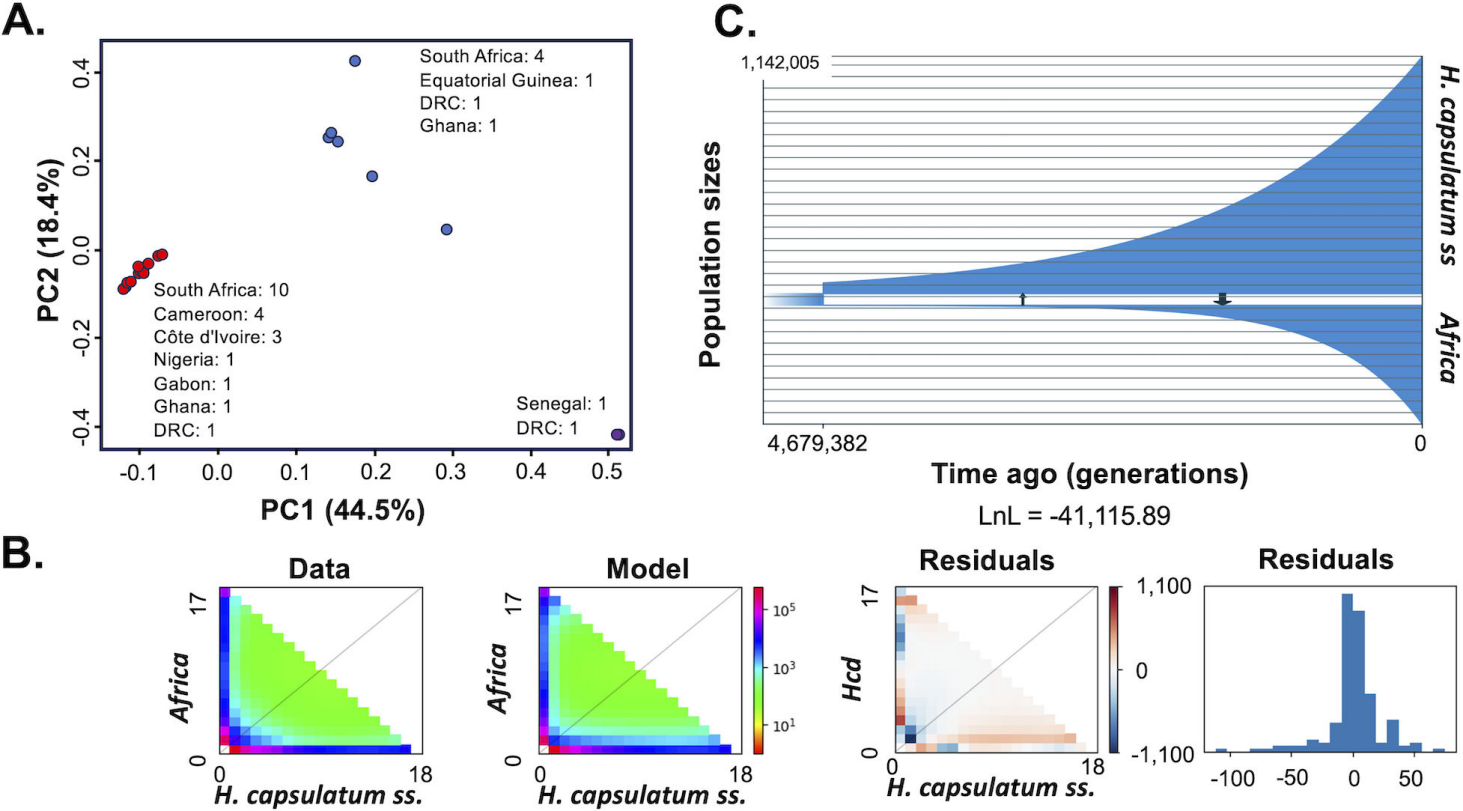
Comparative demography between *Africa* and *H. capsulatum sensu stricto*. (**A**) PCA shows the existence of genetic clusters within *Africa* consistent with population structure not consistent with geographical isolation (see text). Each color represents a cluster. (**B**) Two-species allele frequency spectrum in the data of the two species and in simulated data. Each bin shows the abundance of a combination of allele frequencies in the two species of *Histoplasma*. Abundance is marked by the color scheme in the legend. The two rightmost panels show the residuals between the observed data and the best-fitting simulated data in GADMA as the difference between the two data sets in the allele frequencies per bin and as a histogram of the deviation between models. (**C**) Both Africa and *H. capsulatum sensu stricto* have experienced an effective population size expansion in the last ~4 to 5 million generations. The population expansion is more noticeable in *H. capsulatum sensu stricto*.

Next, we modeled the demographic history of the *Africa* lineage since it split from its sister clade, *H. capsulatum ss*. We found three genetic clusters within this lineage which do not correspond to the geographical location of cases (Fig. 4A). Notably, samples of the three clusters are widespread across the continent. The largest cluster (*n* = 25) shown in red in Fig. 4A includes samples from South Africa, Cameroon, and Côte d'Ivoire. The second cluster (*n* = 7) shown in blue includes samples from South Africa, Ghana, and Equatorial Guinea. The smallest cluster (*n* = 2, shown in purple) includes a sample from Senegal. All three clusters include a sample from the Democratic Republic of Congo. Conscious of this structure, we fit a demographic model to estimate the divergence time between each of these three demes and *H. capsulatum ss*. The results are identical in the three models (one for each of the two largest demes and one for *Africa* altogether). We find the two species have been separated for ~5.2 million generations (Fig. 4B and C). Both species have undergone a rapid increase in population size, but the population growth has been less pronounced in *Africa* than in *H. capsulatum ss*. Note that we did not estimate population size and time since divergence for the *Hcf* lineage because the sample size of this lineage was much smaller, rendering demographic inference challenging.

### The two African lineages (*Africa* and *Hcf*) show signatures of positive selection in their genome

**Fig 5:**
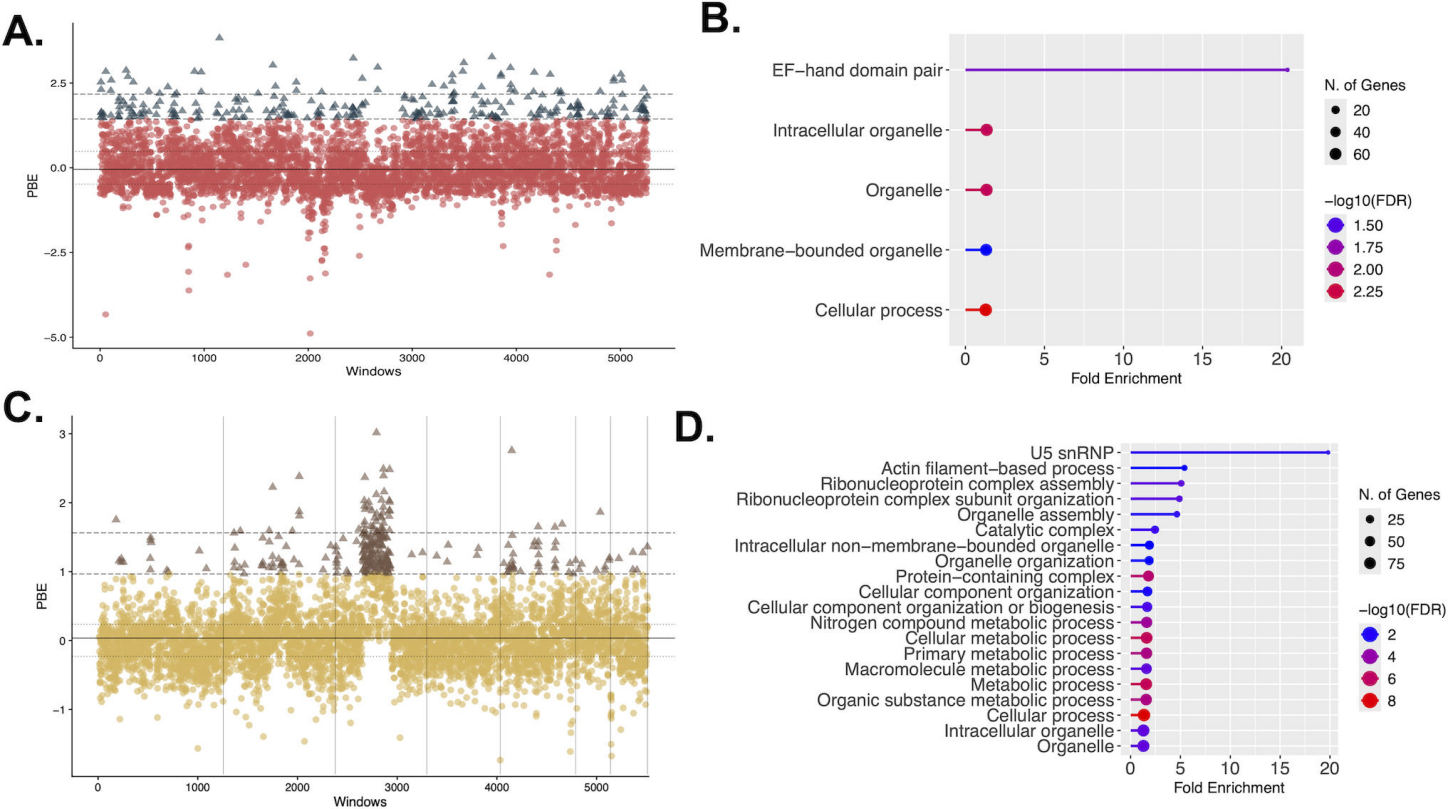
Parallel instances of selection in *Africa* and *Hcf*. (**A**) Population branch excess (PBE) along the genome in *Africa* lineage. We calculated PBE for non-overlapping windows containing 100 SNPs. Windows were 15 kb long on average. (**B**) Gene Ontology analysis for the upper fifth percentile most differentiated genes in *Africa*. (**C**) PBE along the genome in *Hcf*. (**D**) Gene Ontology analysis for the upper fifth percentile most differentiated genes in *Hcf*.

Genes under lineage-specific positive selection often are highly differentiated (72, 74). We hypothesized that there were distinct signatures of selection in the genome of *Africa* and *Hcf*. As expected, the vast majority of the genome shows a high degree of conservation with respect to other species of *Histoplasma* regardless of the metric (Fig. 2B, Dxy; Fig. 5). PBE revealed 512 unique genomic locations under strong selection in *Hcd*, which corresponded to 159 BUSCO genes (Fig. 5A). The gene with the highest PBE value was BUSCO ID: 165at33183, an ortholog of *Saccharomyces cerevisiae* ECM29. The protein has an ECM domain (PFam: PF13001) and seven ARM-like repeats. A second highly differentiated gene with signatures of selection was QSS63771.1 (BUSCO ID: 700at33183), an ortholog of Cdc25p or BUD5. The protein belongs to the Ras guanine nucleotide exchange factor domain superfamily (PANTHER PTHR23113). A Gene Ontology analysis with the genes with homologs in *S. cerevisiae* revealed an enrichment for a single category, EF-hand domain pair (fold enrichment = 20.4, four genes; Fig. 5B).

Similarly, PBE indicated 461 unique genomic locations under strong selection corresponding to 280 BUSCO genes in the *farciminosum* lineage (Fig. 5C). Among the most differentiated genes is *Met18*, which is a key component of the cytosolic iron-sulfur protein assembly (CIA) machinery, involved in the late stages of Fe-S cluster assembly (104, 105). These target proteins play critical roles in processes such as methionine biosynthesis, DNA replication and repair, transcription, and telomere maintenance. In the case of *Histoplasma*, the stoichiometry of copper and these two elements is important for virulence (106, 107). In *Saccharomyces*, *Met18* mutants show decreased sensitivity to cadmium (108); whether met18 is involved in metal tolerance–including copper, and thus in virulence–in *Histoplasma* remains untested. Other genes with high PBE value were QSS65591.1 (BUSCO ID: 4at33183), which corresponds to a dynein heavy chain; *GEA2*, which contains a Mon2/Sec7/BIG1-like, HUS domain (PFAM: pfam PF12783, Interpro:: IPR032691) and a SEC7 domain profile (Interpro: IPR000904); and *PRP8*, which belongs to the PRE-MRNA splicing factor *PRP8* superfamily (PANTHER: PTHR11140). GO analyses suggested that the breadth of genes under selection in *Hcf* was broader than in Africa and indicated an enrichment for genes involved in U5 snRNP (enrichment FDR = 6.0 × 10^−3^, four genes, fold enrichment = 19.8), actin filament-based process (enrichment FDR = 1.2 × 10^−2^, eight genes, fold enrichment = 5.4), spliceosomal conformational changes to generate catalytic conformation (enrichment FDR = 7.9 × 10^−3^, four genes, fold enrichment = 14.9), and two ribonucleoprotein complex assembly processes (enrichment FDR < 1.5 × 10^−3^, 11 genes, fold enrichment = 5.1; Fig. 5D).

Notably, 17 genes show evidence of positive selection in both lineages. Table S5 lists all the loci under positive selection in *Africa* and *Hcf*. Four of these alleles (*PIN4*, *SEC2*, *MET17B*, and *VPS30*) have clear orthologs in *S. cerevisiae*. The size of the intersection is too small to generate GO categories.

### Evidence of introgression

**Fig 6:**
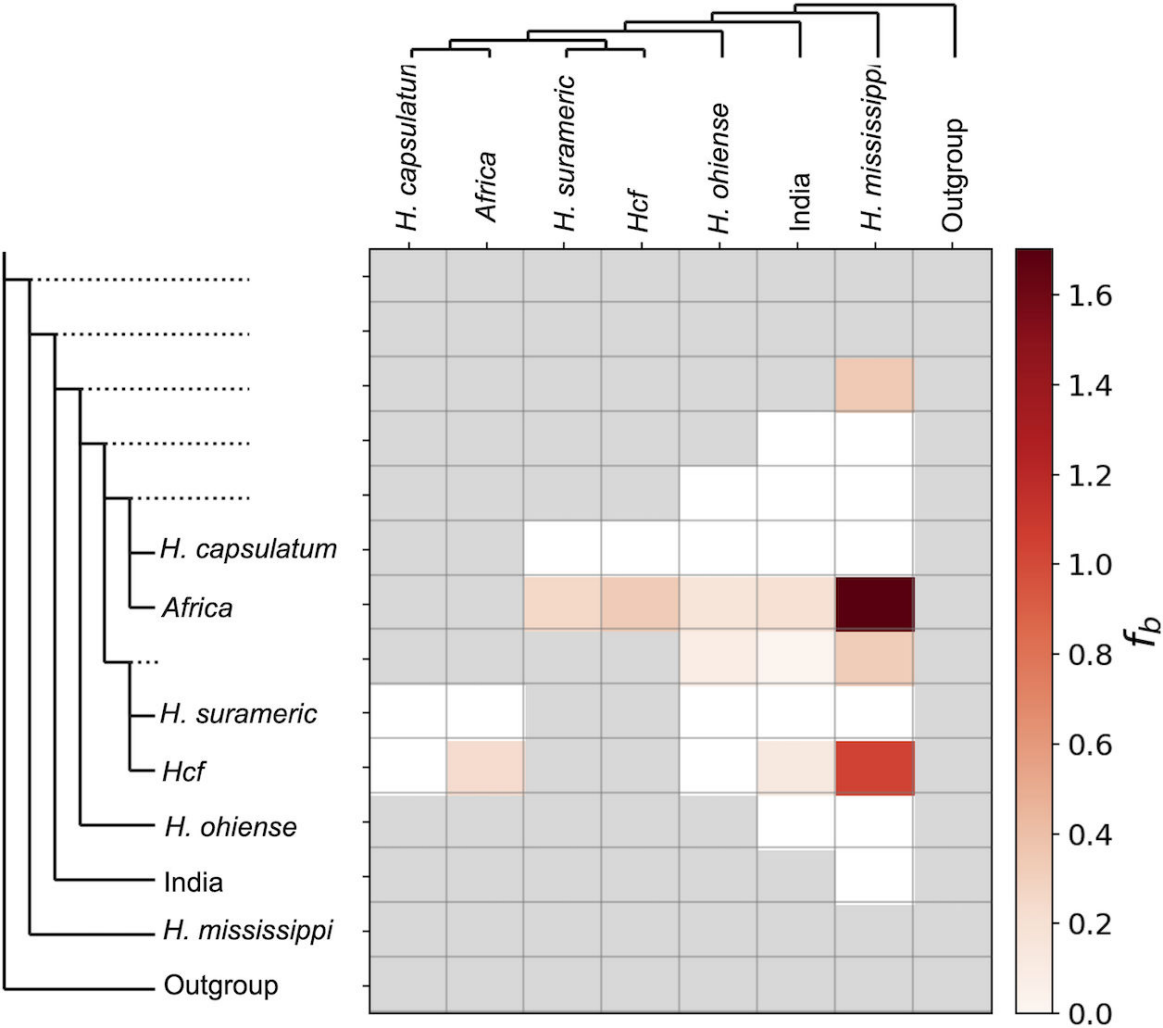
Evidence of introgression in the African species of *Histoplasma*. Fbranch (*f_b_*), based on Patterson’s *D*-statistic, shows excess sharing of derived alleles between the taxa on the *x*-axis and the branches on the *y*-axis. *f_b_* represents the strength of the signal for introgression but is not a percentage. The color of a tile indicates the support for an introgression event between each given pair using *f_b_* as a metric. White tiles show no evidence for introgression (*f_b_* = 0); gray tiles show pairs for which the test could not be performed. Dotted branches symbolize internal branches.

Gene exchange between nascent species has been hypothesized as a potential source of adaptive variation (reviewed in reference 109). The *Africa* and *Hcf* lineages of *Histoplasma* overlap in their geographical range, potentially across Africa. We thus studied whether the *Histoplasma* lineages in Africa exchanged alleles with each other and other related species of *Histoplasma* using genomic data. *f*-branch, a derivative of the *D*-statistics, revealed evidence of gene exchange at low levels. Figure 6 shows the evidence supporting gene flow between different *Histoplasma* species pairs, and Table S6 shows the *D*-statistic results for all trios. The *Africa* lineage shows evidence of introgression with the five species in the analyses, including *Hcf*. The strongest signature of introgression is between *Africa* and *H. mississippiense. Hcf* shows evidence of introgression with three species (*Hcd*, *India*, and *H. mississippiense*) but not with *H. capsulatum ss* or with *H. ohiense*. The evidence of gene flow among *Histoplasma* spp. is comparable with other species of fungi (110). These assessments indicate that there has been a history of introgression that has influenced the phylogenetic history of the African lineages of *Histoplasma*. The strongest signatures of admixture we detected were between *H. mississippiense* and *Africa* lineages, and between *H. mississippiense* and *Hcf*. Note that the *f*-branch is only effective at detecting evidence of gene exchange but does not precisely time the events of introgression and thus is unable to exactly pinpoint the species involved in the admixture events.

### Epidemiological patterns of African histoplasmosis

We compiled the available clinical data for the isolates reported here and used these data to compare four key population attributes of the disease (skin involvement, patient sex, patient age, and HIV infection status). We compared these facets between histoplasmosis caused by the *Africa* lineage and the disease caused by five other *Histoplasma* spp. No human clinical cases were found for the *Hcf* clade, so we restricted these analyses to the *Africa* lineage for which we had human patient clinical histories. Three of the archived South African isolates had no linked clinical data. A summary of clinical characteristics for 17 cases (13 South Africa, 4 Spain) is shown in Table 1.

First, we examined histoplasmosis skin involvement across different lineages of *Histoplasma* (Table S7). The proportion of patients with skin involvement is higher in histoplasmosis caused by *Africa* than in histoplasmosis caused by *H. capsulatum ss*, mz5-like, or *H. ohiense* (*P* < 0.0351, Table S7) but does not differ from *H. suramericanum* or *H. mississippiense* (*P* = 0.447 and *P* = 0.242, respectively). Note that the power of these two latter tests is limited (power <0.427). This result might represent an ascertainment bias as skin symptoms are among the main indicators prompting diagnosis of African histoplasmosis. Next, we studied whether there was a difference in the proportion of histoplasmosis cases who were living with HIV among six different phylogenetic species of *Histoplasma*. The proportion of patients living with HIV was lower in histoplasmosis caused by the North American species of *Histoplasma* (*H. mississippiense* = 0.333, *H. ohiense* = 0.588) than in histoplasmosis caused by any of the other species–including *Africa*—all of which showed a proportion of coinfection close to 100%. Again, ascertainment bias is an issue since in Africa, advanced HIV disease can be a prompt to consider disseminated histoplasmosis as a diagnosis. Table S8 shows the pairwise comparisons between different phylogenetic species. Third, we studied whether there was a difference in the proportional representation of patient sex in histoplasmosis caused by *Africa* and five more *Histoplasma* phylogenetic species. *Africa* showed the highest proportion of female patients (50%), and *H. capsulatum ss.* showed the lowest proportion (6.7%). Note that our current sample has little power to detect pairwise differences in sex ratio among phylogenetic species (Table S9). Finally, patient age at diagnosis showed differences among phylogenetic species (F_2,104_ = 2.398, *P* = 0.021) but none of the pairwise comparisons involving the *Africa* lineage were significant (Table S10).

### Geographical range determinants of African *Histoplasma*

**Fig 7:**
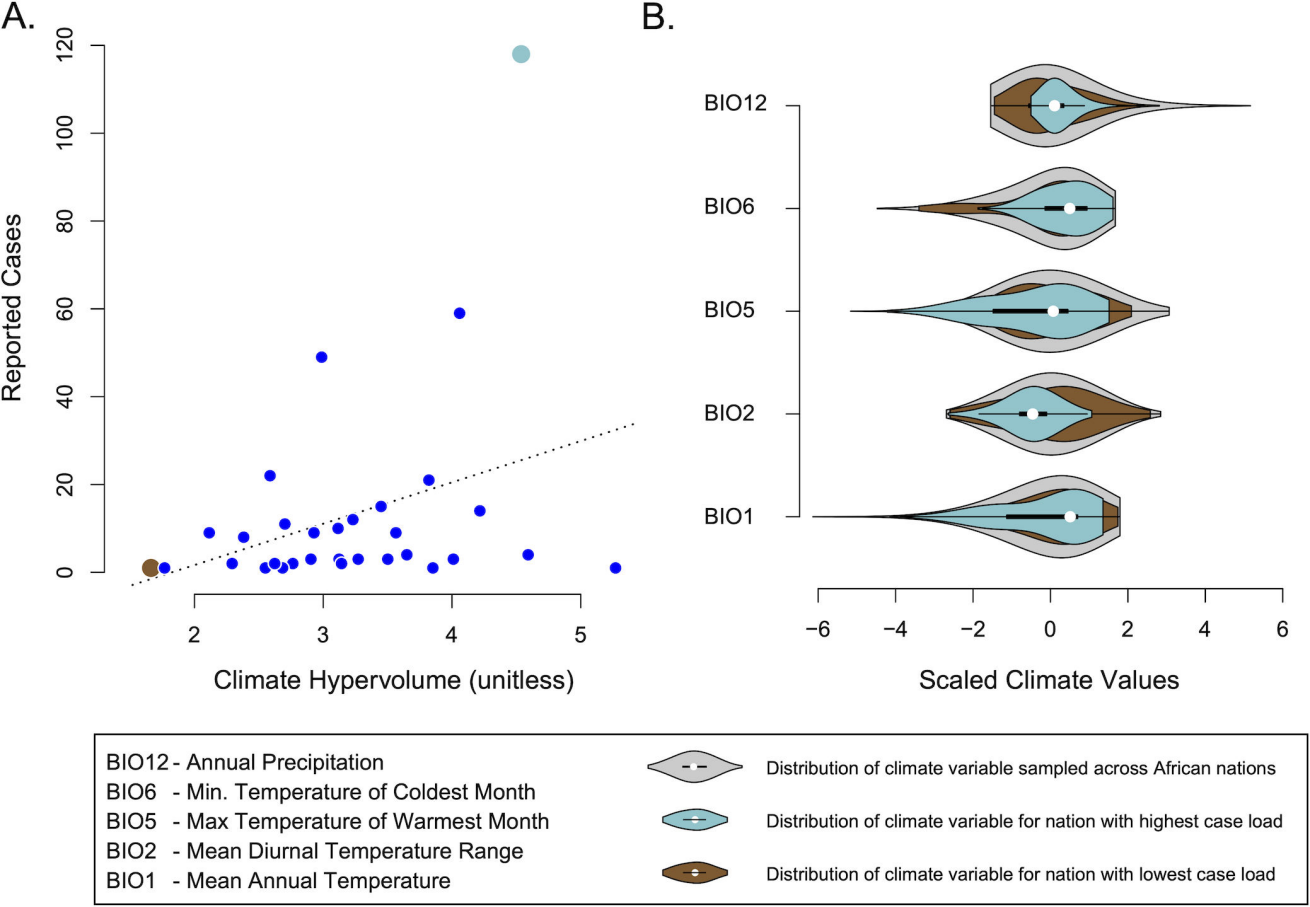
Bioclimatic niche characterization of African histoplasmosis. (**A**) The national caseload among African countries is weakly predicted (*P* = 0.067) by the climatic breadth of the country. National climatic niche distributions were modeled by randomly sampling 1,000 geographical points within each country’s borders and extracting data for the points from the WorldClim database (2.5 arcminute resolution). We modeled climatic breadth in each country using multivariate hypervolumes, including temperature and precipitation, along with their diel and seasonal fluctuations. (**B**) There was broad overlap among the countries with the largest and smallest caseloads on all climatic niche axes. Gray distributions depict the pooled climatic niche across all countries for which we have case data. The brown distributions show the climate niche of the country with the lowest caseload, contrasting with the blue distributions that depict the climatic breadth of the country with the highest caseload.

The *Africa* samples in this study come from a diverse sample of countries in Africa (see Table S1; Fig. 4). We hypothesized that the lineage might be widely distributed in Africa but that it might be constrained by its climatic niche. The available metadata for African histoplasmosis cases does not include the particular locality of the infected individuals and is largely restricted to caseload by country. Because of this, rather than directly characterizing the climatic breadth of observed cases, we calculated the climatic breadth for each of the countries for which we have caseload data (14, 88). We present these results as multidimensional hypervolumes (*n*-dimensional spaces) where each dimension is one of the climatic axes. We tested whether climate predicts the prevalence of histoplasmosis and found that there was substantial variation among countries in the breadths of their climate hypervolumes (variance = 22.9% of the mean; Fig. 7A; Fig. S7). This result demonstrates that, not surprisingly, climate varies considerably among African countries. The broadest climatic niche was South Africa (hypervolume = 5.33), and the narrowest was the Central African Republic (hypervolume = 1.63). Because these countries also vary in their land area and, intuitively, larger areas can encompass more diverse habitats and conditions, we tested whether land area was a predictor of climate niche breadth. We find no significant relationship between niche hypervolume and land area (*F*_1,29_ = 0.786, *P* = 0.383). We also quantified the distributions of each of the constituent climate axes, and while the distributions overlap strongly (see Fig. 7B), we find variation in their breadth (measured as their middle 95% quantile range; Fig. S11 and S12). Finally, we used generalized linear models to test whether climate hypervolume predicts the caseload of histoplasmosis and found that climate loosely predicts the incidence of infection (*F*_1,29_ = 4.129, *P* = 0.0514; Fig. 7A; see also Fig. S11). The individual climate variables were not predictive of caseload (all *P* > 0.30). Similarly, the breadth of each climate axis was not predictive of caseload (most *P* > 0.15), though we did find a trend between mean diurnal temperature range and caseload (*P* = 0.075). This relationship is non-significant, so while we note the trend, we hesitate to interpret further. The complete results of our regression analyses are detailed in Fig. S11.

**Fig 8:**
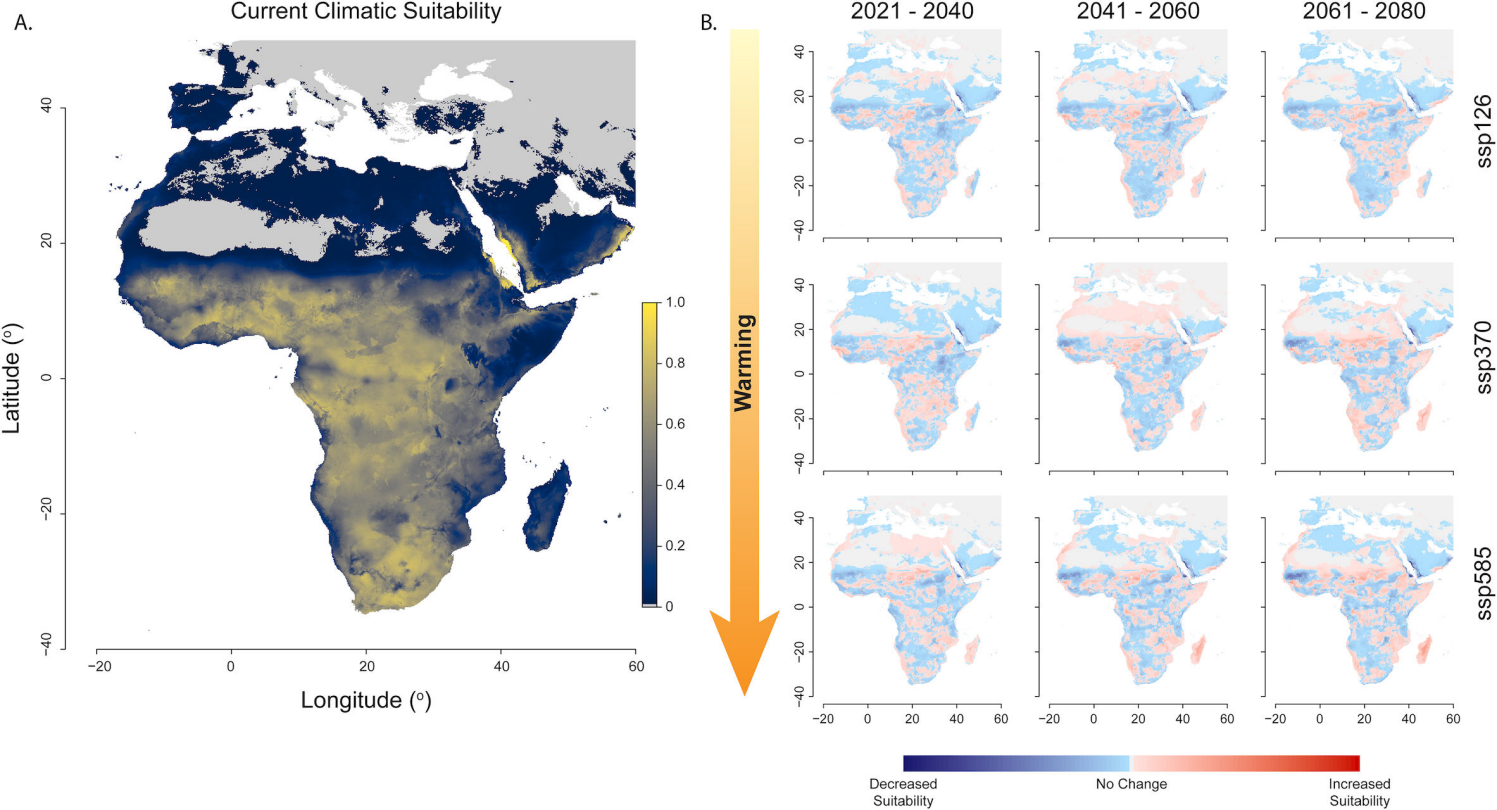
Modeling current and future climatic suitability of African histoplasmosis cases. (**A**) Bioclimatic niche modeling suggests that climatic conditions are suitable for the presence of histoplasmosis across most of the African continent, with some regions (in yellow) of high climatic suitability (>0.6 in the MaxENT model, 6.5% of land area). The majority of land area in Africa is moderately suitable to support *Histoplasma* (between 0.4 and 0.6, 83.1%), with less land area being of low suitability (<0.4, 15%). (**B**) Projections of climatic suitability across three models of climatic warming show that climate will remain broadly suitable across Africa, but that the geographical distribution of climatic suitability will shift across the continent. Increases in climatic suitability are shown in red; decreases are shown in blue. Climate suitability models are shown in Fig. S8.

Next, we used climate conditions across the African continent to predict the risk of *Histoplasma* infection using MaxENT modeling. MaxENT modeling is a machine learning technique that pairs species observation data with geographical distribution of climatic conditions to predict species distribution. We constructed a MaxENT model based on the climate conditions at 1,000 random geographical locations taken from the countries with the three highest caseloads. Using this model, we projected the geographical distribution and degree of suitability of climate conditions for histoplasmosis caused by the Africa lineage across the region (Africa, the Middle East, and southern Europe; see Fig. 8A). Based on this model, we find that 95.4% of the modeled region is currently suitable for the development of histoplasmosis (Fig. 8A). To understand the magnitude of the risk, we defined high risk as climatic suitability of >0.6, moderate risk as 0.4–0.6, and low risk as <0.4 and recorded the proportion of land area in each projection that falls into each of these bins. We find that 6.5% of the modeled area falls into the high-risk category, 83.1% into the moderate-risk range, and 10.4% into the low-risk range. Only 4.6% of the study region is entirely outside of the predicted climate envelope (climate suitability <0.01 in our MaxENT model) for histoplasmosis. The geographical extent of moderate to high climate suitability suggests that cases may be more widespread than currently recognized (Fig. 8A).

Finally, we used three projections of future climate conditions, with increasing levels of anthropogenic warming, to predict how the risk of histoplasmosis might shift across the African continent. To better understand the human health risk that *Histoplasma* will pose as climate change progresses, we used three climate projections (mild, moderate, and extreme warming) to model how the regions of suitable climate conditions (based on current conditions, described immediately above) will shift across the region in three future time ranges (Fig. S7 and S8B). In all three warming regimes, the mean climatic suitability across the African continent is projected to increase sharply in the 2021–2040 interval (between 22% and 23%, depending upon climate projection vs 15.77% in current conditions; see Fig. 8; Table S13, also Table S7 and S8). Mean climatic suitability is projected to decrease from the initial maximum across the two subsequent intervals (2041–2060 and 2061–2080; Fig. 8B; Table S13), with the mild warming scenario maintaining the greatest mean suitability, the extreme warming scenario decreasing the most, and the moderate warming scenario being intermediate. All three projections across all modeled time intervals are projected to have greater climatic suitability for histoplasmosis infections to occur than our model predicts for current climate conditions. Further, the proportion of land in the high and moderate risk categories increased across all projected intervals (see Table S13). Notably, the distribution of risk is projected to shift across the landscape, following favorable climate and highlighting the need for enhanced vigilance in regions where histoplasmosis has not been a historic challenge (see Fig. 7).

## DISCUSSION

Genomics has revolutionized our understanding of biological diversity, and fungal pathogens have not been an exception to these insights. Phylogenomics has led to an extensive taxonomic revision in fungal pathogens causing human disease (51, 111, 112); the genus *Histoplasma* contains at least six phylogenetic species (28). These newly recognized species differ in their morphology (29) and, in some cases, virulence traits (30). In this piece, we report the existence of two better-sampled lineages of *Histoplasma* in Africa, one of which is often associated with skin manifestations of histoplasmosis (*Africa*) and another that seems to be associated with epizootic lymphangitis in equines (*Hcf*). This survey constitutes the largest genomic epidemiology effort to characterize African histoplasmosis to date. We also provide evidence that African histoplasmosis is likely to expand its current geographical range due to global warming.

The main overarching result of this piece is that we show evidence of the existence of two main lineages of *Histoplasma* in Africa that fulfill the characteristics to be considered phylogenetic species: *Africa* and *Hcf*. Initial taxonomic analysis using morphological and histological methods had proposed the existence of different lineages of *Histoplasma* in Africa (113). *Hcd* induces a form of histoplasmosis, known as African histoplasmosis, which is characterized by extensive tissue destruction and the formation of large granulomas (15, 16, 20). *Africa* and isolates typically classified as *Hcd* seem to be the same genetic group, but we do not equate the two names here because we are unable to conclude that *Africa* is the only lineage that causes African histoplasmosis. Our results also suggest that histoplasmosis caused by the *Africa* lineage shows frequent skin involvement. Nonetheless, this result might be the result of an ascertainment bias in which skin histoplasmosis is diagnosed more readily than the pulmonary form. Whether *Africa* is the only species of *Histoplasma* that causes African histoplasmosis (i.e., are *Africa* and *Hcd* the same lineage?), the symptomatology of African histoplasmosis will require longitudinal follow-up of patients and genetic characterization of isolates causing pulmonary histoplasmosis in Africa.

Our results also revealed that the *Africa* lineage is genetically diverse. While several genomic assessments have indicated the existence of this lineage (24, 28, 31), the low representation of genomic sequences had suggested a clonal nature of *Histoplasma* in Africa (28). Our assessments indicate this is not the case and that, as a matter of fact, demographic analyses revealed that the *Africa* clade has undergone an effective population size increase in the recent past. Currently, the *Africa* lineage harbors a level of diversity comparable with the other species of *Histoplasma* (Fig. 2A, diagonal) and other fungal species (114). This suggests that the previous assessments of the magnitude of genetic variability of *Histoplasma* in Africa were limited by their sample size. *Africa* shows evidence of population structure not necessarily associated with a clear geographical pattern and evidence of introgression with other species of *Histoplasma*. Altogether, the population structure, the increase in effective population size, and the influx of introgressed haplotypes might serve as the raw material to adaptation for range expansion as climate changes. The underlying causes of population structure within this lineage, the evolutionary processes that have driven the evolution of *Africa*, the extent of diversity, and the sources of genetic variation in *Africa* and other African lineages of *Histoplasma* are questions that remain unexplored, and answering them will require additional population sampling.

The inclusion of three equine-derived samples also revealed that the three veterinary samples form a monophyletic group consistent with the existence of another classically defined lineage of *Histoplasma*, *Hcf*. Two previous surveys (1, 115) used MLST to study the genetic relationships between veterinary and clinical samples of *Histoplasma*. The former used exclusively donated equine-derived samples, and the latter followed a veterinary survey on a variety of mammals (cats, badgers, mice, horses, dogs, hedgehogs, and gazelles). Both studies concluded that the animal samples are polyphyletic as they are interspersed with clinical isolates of *Histoplasma* (115). These previous results and ours are not directly comparable because the sampling structure in terms of isolates and loci differs from each other. Two possibilities can explain this apparent discordance. First, we identified a monophyletic lineage of *Histoplasma* causing disease in equines, but that does not preclude the possibility that other lineages can also cause epizootic lymphangitis in animals, which would manifest as a polyphyletic clade. Second, the two types of analyses, our phylogenomic tree and the previous MLST, both have limitations. Despite genomic coverage, our sample size is small, and our assessment cannot be compared with a deeper sampling but typed with MLST. The use of MLST for phylogenetic analyses is limited as the approach can only reveal the gene genealogies from the loci in the study and not the species tree. The causes behind these differences can be addressed by building phylogenetic trees that use whole-genome data from more samples and ideally with a wider geographical sampling (see below).

We refer to the two lineages reported here as African *Histoplasma* lineages, but it is possible that the geographical range of the two lineages is larger than what we report here. Current genomic sampling suggests that histoplasmosis caused by *Africa* seems to be restricted to the African continent, but we included cases in patients with prior residence in Africa but diagnosed in Europe. Previous studies have also reported genotypes consistent with *Hcd* in Spain (116, 117). Ten patients diagnosed with histoplasmosis between 1997 and 2014 had a disease consistent with the one caused by *Hcd*, but no travel history for these patients is known (116). Similarly, veterinary surveys have found epizootic lymphangitis caused by *Histoplasma*, which is consistent with *Hcf*—or a convergent lineage—in other places outside of Africa such as the Middle East (118, 119), Poland (1), and Germany (115, 120, 121). Histoplasmosis in horses in North America differs from the classical epizootic lymphangitis and induces granulomatous placentitis and abortion (122). These reports predate genome sequencing, and these cases await genomic assessment. Determining whether the ability to cause epizootic lymphangitis in horses and other animals evolved once in *Histoplasma* or multiple times (as proposed in references 1 and 123) will require sampling around the world and across diverse host species; our current sampling does not have that level of granularity.

Evolutionary genomics has other uses besides the identification of species boundaries. Our genomic scans also revealed alleles that have evolved through positive natural selection in each of the two African lineages. In the *Africa* lineage, *ECM29* is one of the most differentiated genes (Fig. 5). Mutant alleles of this gene in *S. cerevisiae* cause differential resistance to chemicals, such as paromomycin (124), calcofluor white (125), and hygromycin (125). An additional finding that is worth pursuing is the enrichment for EF-hand domain-containing proteins in the genes under selection in the *Africa* lineage. This family of proteins binds calcium, plays a key role in calcium signaling, and has been involved in virulence in prokaryotic and eukaryotic pathogens (reviewed in reference 126). In *Toxoplasma*, disruptions of EF-hand domain-containing proteins led to a slower growth rate (127). In *Histoplasma*, the calcium-binding protein (CBP) has been conclusively demonstrated to be involved in virulence (128, 129). A second protein, BAD1, is involved in virulence in the closely related fungal pathogen *Blastomyces* (130–133). Calcineurin, a phosphatase that is dependent on calcium and calmodulin signaling, is a conserved protein across eukaryotes which has been implicated in virulence pathways across eukaryotic pathogens (reviewed in reference 134).

The *Hcf* lineage also shows distinct signatures of selection. *PRP8* in *S. cerevisiae* has been regularly implicated in thermal fitness (135). A second example, the homolog of *GEA2* in *S. cerevisiae*, a *SEC7* domain-containing protein, is highly differentiated in the *Hcf* lineage. Mutant alleles in *S. cerevisiae* show decreased resistance to a variety of chemicals such as nocodazole (136), hydroxyurea (137), and bleomycin (137). None of these compounds is currently used for antifungal treatment. Perhaps more notable, mutant alleles of *GEA2* are also temperature sensitive, suggesting this protein might play a role in thermal fitness (138). Given the thermal dimorphism of *Histoplasma* and that thermotolerance is an important fitness factor in *Histoplasma* (139), both *PRP8* and *GEA2* in the *Hcf* lineage are alleles of interest. Functional work should be pursued to determine whether these alleles are involved in the different symptoms that the two African lineages of *Histoplasma* reported here cause in equines and human patients.

Notably, we detected 17 parallel instances of selection in *Africa* and *Hcf*. All these genes show phenotypes of interest in yeast that could be implicated in virulence in *Histoplasma. SEC2* in yeast has alleles that are thermosensitive (140) and is involved in increased heat sensitivity. Some alleles of *VPS30* show increased tolerance to metals (Zn, Co, and Ni [108, 141]), while others show decreased tolerance to metals (Ca, Al, and Gd [108, 142, 143]) or metalloids (As [108]). Similarly, null alleles of *PIN4* show decreased resistance to metals (Ca, Ga, Al, and In [108, 143]). Finally, alleles of *MET17B* show increased metal resistance to methylmercury and calcium chloride (144, 145).

The presence of an isolate from the mz5-like lineage in Africa points to limitations on the current understanding of the geographical range of *Histoplasma* species. Two possibilities could explain this pattern. The first possibility is that histoplasmosis in this patient was acquired in the known geographical range of this lineage, South America. We are unable to address this possibility as we do not have information on the patient travel history. The second possibility is that the mz5 lineage is one of the causal agents of classical histoplasmosis in Africa and that the range of this species is larger than previously thought. These two hypotheses are not mutually exclusive. Determining the geographical range of the different *Histoplasma* spp. and the extent of migration of *Histoplasma* in patients with histoplasmosis is urgent for all the described phylogenetic species (e.g., see reference 146). Our focus in this study is to understand the isolates that cause African histoplasmosis and thus has limited power to inform on the evolutionary history of the clinical agents of classical histoplasmosis in Africa.

Our bioclimatic niche modeling suggests that *Histoplasma* and associated cases of histoplasmosis may be more widespread across the continent than previously thought and that the magnitude of the histoplasmosis threat will shift across the landscape tracking future climatic changes. These results underscore the need to improve clinical screening and diagnostic capabilities across Africa, especially as the planet continues to warm. More broadly, climate change has been implicated as a driver of increasing, emerging, and shifting human health threats from a variety of pathogens (e.g., see references 147–150), but fungal pathogens may be especially sensitive to climatic conditions (151–153). This includes *Histoplasma* and other thermally-dimorphic fungal pathogens. Increasing local mean temperatures and more frequent flooding events are strongly associated with a rise in fungal infections, particularly those with cutaneous presentations, at increasingly higher latitudes (151, 152, 154).

MaxENT modeling is an effective approach for assessing climatic tolerance and forecasting geographical distribution influenced by climate; however, our implementation here is novel and not without caveats. MaxENT is robust to the use of presence-only data (155); however, the geographical points that we used to train our model were not actual case localities, which would be the ideal situation. Because most cases are reported from cities with advanced hospital facilities, or as a per-country count, we based our model on the breadth of climate conditions within the three countries having the highest caseloads under the assumption that the conditions within these countries represent the greatest climatic suitability for infection. It remains possible that these countries do not represent the actual climatic breadth of the pathogen and may be the result of reporting bias arising from either heightened vigilance within those countries or underreporting elsewhere. We stress that location data or place of residence should be reported alongside positive diagnoses to facilitate future climatic studies of African histoplasmosis.

In summary, in this report, we integrate clinical sampling, evolutionary genomics, and environmental niche modeling to study multiple facets of the biology of the etiological agents of African histoplasmosis. The approaches and tools presented here can serve as a blueprint to understand unexplored aspects of the biology of neglected pathogens.

## SUPPLEMENTAL MATERIAL

Supplemental material mbio.00564-25-s0001.docx Tables S1-S13; Figures S1-S8.

ASM does not own the copyrights to Supplemental Material that may be linked to, or accessed through, an article. The authors have granted ASM a non-exclusive, world-wide license to publish the Supplemental Material files. Please contact the corresponding author directly for reuse.
