# Supplementary material for: Genomic epidemiology of Histoplasma in Africa
Source: mBio. 2025 Aug 5;16(9):e00564-25. doi: 10.1128/mbio.00564-25 (PMC12421976; doi:10.1128/mbio.00564-25)
Supplement: Supplemental material — Tables S1-S13; Figures S1-S8. [file mbio.00564-25-s0001.docx]

**Genomic epidemiology of Histoplasma in Africa. SUPPLEMENTARY MATERIAL.**

**TABLES S1-S13**

**FIGURES S1-S8**

**SUPPLEMENTARY TABLES**

**TABLE S1.** Isolate information for all the newly reported isolates. The last column shows the SRA accession number for the genome data.

|  | **Name** | **filename** | **bioproject_accession** | **accession** | **Country of origin** | **Other names** |
| --- | --- | --- | --- | --- | --- | --- |
| 1 | T-3-1 | SA15_S82_L001_R1_001.fastq.gz | PRJNA1109714 | SRR29905427 | Archive sample in South Africa | ATCC 22635 |
| 2 | 19VMG-15 | 19VMG-15_S22_L001_R1_001.fastq.gz | PRJNA1109714 | SRR29905426 | South Africa | - |
| 3 | 20VMK11 | 20VMK11_S8_R1_001.fastq.gz | PRJNA1109714 | SRR29905415 | South Africa | - |
| 4 | CM7001 | HISSP-CM7001-xx-CL-GNQ-xxxx-036-BB_S14_R1_001.fastq.gz | PRJNA1109714 | SRR29905419 | Equatorial Guinea | - |
| 5 | CM7434 | HISSP-CM7434-xx-CL-ZAF-xxxx-036-BB_S18_R1_001.fastq.gz | PRJNA1109714 | SRR29905418 | Senegal | - |
| 6 | CM7704 | HISSP-CM7704-xx-CL-GHA-xxxx-036-BB_S28_R1_001.fastq.gz | PRJNA1109714 | SRR29905417 | South Africa | - |
| 7 | CM7767 | HISSP-CM7767-xx-CL-NGA-xxxx-036-BB_S23_R1_001.fastq.gz | PRJNA1109714 | SRR29905416 | Ghana | - |
| 8 | CM8013 | HISSP-CM8013-xx-CL-GHA-xxxx-036-BB_S24_R1_001.fastq.gz | PRJNA1109714 | SRR29905414 | Nigeria | - |
| 9 | CM5788 | HISSP-CM5788-xx-CL-xxx-xxxx-036-BB_S6 | PRJNA1109714 | SRR33700783 | unknown | - |
| 10 | ES2_82 | ES2_82_S60_R1_001.fastq.gz | PRJNA1109714 | SRR29905413 | Ivory Coast | - |
| 11 | ES2_83 | ES2_83_S61_R1_001.fastq.gz | PRJNA1109714 | SRR29905412 | Africa | - |
| 12 | ES2_84 | ES2_84_S63_R1_001.fastq.gz | PRJNA1109714 | SRR29905411 | Republic of Cameroon | - |
| 13 | ES2_85 | ES2_85_S64_R1_001.fastq.gz | PRJNA1109714 | SRR29905410 | Republic of Cameroon | - |
| 14 | ES2_86 | ES2_86_S66_R1_001.fastq.gz | PRJNA1109714 | SRR29905409 | Republic of Cameroon | - |
| 15 | ES2_87 | ES2_87_S68_R1_001.fastq.gz | PRJNA1109714 | SRR29905408 | Democratic Republic of Congo | - |
| 16 | ES2_88 | ES2_88_S70_R1_001.fastq.gz | PRJNA1109714 | SRR29905407 | Republic of Cameroon | - |
| 17 | ES2_89 | ES2_89_S72_R1_001.fastq.gz | PRJNA1109714 | SRR29905406 | Democratic Republic of Congo | - |
| 18 | ES2_90 | ES2_90_S74_R1_001.fastq.gz | PRJNA1109714 | SRR29905405 | Ivory Coast | - |
| 19 | ES2_91 | ES2_91_S76_R1_001.fastq.gz | PRJNA1109714 | SRR29905403 | Gabonese Republic | - |
| 20 | CBS 536.84 | HISSP-CM7436-xx-CL-EGY-xxxx-036-BB_S20_R1_001.fastq.gz | PRJNA1109714 | SRR29905402 | Egypt | CM7436 |
| 21 | B05181 | HISSP-B05181-xx-CL-CIV-xxxx-036-BB_S63_R1_001.fastq.gz | PRJNA1109714 | SRR29905420 | Ivory Coast | - |
| 22 | SA4 | Low DNA quality. Not analyzed. | | | South Africa | - |
| 23 | SA46 | 46SS_S9_R1_001.fastq.gz | PRJNA1109714 | SRP521167 | South Africa | - |
| 24 | SA47 | 47B_S50_L001_R1_001.fastq.gz | PRJNA1109714 | SRR29905424 | South Africa | - |
| 25 | SA19 | SA19_S187_L001_R1_001.fastq.gz | PRJNA1109714 | SRP521167 | South Africa | - |
| 26 | SA297 | 297-CGG_S1_L001_R1_001.fastq.gz | PRJNA1109714 | SRR29905397 | South Africa | - |
| 27 | SA302 | 302FLL_S5_R1_001.fastq.gz | PRJNA1109714 | SRR29905396 | South Africa | - |
| 28 | SA355 | 355UP_S51_L001_R1_001.fastq.gz | PRJNA1109714 | SRR29905395 | South Africa | - |
| 29 | SA562 | SA562_S53_L001_R1_001.fastq.gz | PRJNA1109714 | SRR29905421 | South Africa | - |
| 30 | SA602 | 602-TD_S3_L001_R1_001.fastq.gz | PRJNA1109714 | SRR29905423 | South Africa | - |
| 31 | SA811 | 811-ND_S4_L001_R1_001.fastq.gz | PRJNA1109714 | SRR29905422 | South Africa | - |
| 32 | SA1356 | Low DNA quality. Not analyzed. | | | South Africa | - |
| 33 | SA1436 | 1436BN_S7_R2_001.fastq.gz | PRJNA1109714 | SRR29905399 | South Africa | - |
| 34 | SA1371_S188 | 1371NJ_S6_R1_001.fastq.gz | PRJNA1109714 | SRP521167 | South Africa | - |
| 35 | SA1556 | 1556-ZM_S6_L001_R1_001.fastq.gz | PRJNA1109714 | SRR29905398 | South Africa | - |
| 36 | SA1704 | 1704DA_S44_L001_R1_001.fastq.gz | PRJNA1109714 | SRR29905404 | South Africa | - |

**TABLE S2.** Accession numbers for all previously sequenced genomes of Histoplasma used in this study.

| **Isolate name** | **SRA accession number** | **Species** |
| --- | --- | --- |
| CI_24 | SRX3350845 | *H. mississippiense* |
| CI_43 | SRX3350837 | *H. mississippiense* |
| CI_22 | SRX3350842 | *H. mississippiense* |
| CI_7 | SRX3350840 | *H. mississippiense* |
| CI_42 | SRX3350844 | *H. mississippiense* |
| 505 | SRX3350830 | *H. mississippiense* |
| DOWNS | SRX3350816 | *H. mississippiense* |
| CI_19 | SRX3350843 | *H. mississippiense* |
| WU24 | SRX3350838 | *H. mississippiense* |
| UCLA-531 | SRX3350836 | *H. mississippiense* |
| Hc1986 | [SRR6243651](https://trace.ncbi.nlm.nih.gov/Traces?run=SRR6243651) | *H. ohiense* |
| G222B | SRX3350818 | *H. ohiense* |
| G217B | SRX3350817 | *H. ohiense* |
| CI_10 | SRX3350821 | *H. ohiense* |
| CI_4 | SRX3350841 | *H. ohiense* |
| CI_17 | SRX3350822 | *H. ohiense* |
| CI_9 | SRX3350820 | *H. ohiense* |
| CI_30 | SRX3350824 | *H. ohiense* |
| CI_18 | SRX3350823 | *H. ohiense* |
| CI_6 | SRX3350819 | *H. ohiense* |
| CI_35 | SRX3350825 | *H. ohiense* |
| 104_p_06_S19 | SRR27481878 | India |
| 104_P_19_S5 | SRR27481877 | India |
| 107_P_06_S1 | SRR27481870 | India |
| 117_p_12_S17 | SRR27481869 | India |
| 122_p_10_B_S15 | SRR27481868 | India |
| 136_P_07_S6 | SRR27481867 | India |
| 144_p_08_S14 | SRR27481866 | India |
| 1517_p_17_S20 | SRR27481865 | India |
| 256_P_18_S2 | SRR27481864 | India |
| 316_p_10_S18 | SRR27481863 | India |
| 327_P_12_S7 | SRR27481876 | India |
| 343_p_18_S1 | SRR27481875 | India |
| 388_p_11_S16 | SRR27481874 | India |
| Dr_Anuradha_Fungal_WGS_S11 | SRR27481873 | India |
| WGS_S14 | SRR27481872 | India |
| WGS_S16 | SRR27481871 | India |
| HcMV3 | SRX3350826 | *H. capsulatum ss* |
| G186A | SRX3350828 | *H. capsulatum ss* |
| 1014-Belem3 | PRJNA1205708 | *H. capsulatum ss* |
| 2363-Belem6 | PRJNA1205708 | *H. capsulatum ss* |
| 3865-Belem8 | PRJNA1205708 | *H. capsulatum ss* |
| 4182-Belem9 | PRJNA1205708 | *H. capsulatum ss* |
| 4809-Belem13 | PRJNA1205708 | *H. capsulatum ss* |
| 52292-Belem19 | PRJNA1205708 | *H. capsulatum ss* |
| B05368 | PRJNA1205708 | *H. capsulatum ss* |
| B05593-Histo79 | PRJNA1205708 | *H. capsulatum ss* |
| CM6408 | PRJNA1205708 | *H. capsulatum ss* |
| CM6556 | PRJNA1205708 | *H. capsulatum ss* |
| FGDOS0487 | SRR31893277 | *H. capsulatum ss* |
| FGMARTIN | SRR31893276 | *H. capsulatum ss* |
| FGSAT2037 | SRR31893275 | *H. capsulatum ss* |
| FGWIL2021 | SRR31893274 | *H. capsulatum ss* |
| GJJ-CL-GUY | SRR31893273 | *H. capsulatum ss* |
| SROB2039 | SRR31893272 | *H. capsulatum ss* |
| STAN2075 | SRR31893271 | *H. capsulatum ss* |
| Histo_hc3645 | SRR31893270 | *H. capsulatum ss* |
| G184A | SRX3350831 | *H. capsulatum ss* |
| HcMZ5 | SRX3350829 | mz5-like |
| Belem1 | PRJNA1205708 | Amazon-I |
| Belem2 | PRJNA1205708 | Amazon-I |
| Belem5 | PRJNA1205708 | Amazon-I |
| Belem7 | PRJNA1205708 | Amazon-I |
| Belem10 | PRJNA1205708 | Amazon-I |
| Belem17 | PRJNA1205708 | Amazon-I |
| Belem16 | PRJNA1205708 | Amazon-I |
| Belem14 | PRJNA1205708 | Amazon-I |
| 27_14 | SRX3350832 | *H. suramericanum* |
| 3_11G | SRX3350833 | *H. suramericanum* |
| 21_14 | SRX3350835 | *H. suramericanum* |
| CM7717 | SRR31893262 | *H. suramericanum* |
| HC7072a | SRR31893261 | *H. suramericanum* |
| HC7090 | SRR31893260 | *H. suramericanum* |
| SDIJ2058 | SRR31893259 | *H. suramericanum* |
| Histo | SRR31893258 | *H. suramericanum* |
| Histo_HC3066 | SRR31893256 | *H. suramericanum* |
| Histo_HC394 | SRR31893255 | *H. suramericanum* |
| Histo_HC4137 | SRR31893254 | *H. suramericanum* |
| Hc_duboisii-A | SRX3350834 | Africa |
| Hc_duboisii-B | SRX3350839 | Africa |

**TABLE S3.** Accession numbers for all previously sequenced genomes used in this study.

| **Samples** | **SRA** | **Species** | **Reference** |
| --- | --- | --- | --- |
| 109_P_06_S4 | PRJNA1201237 | *Blastomyces dermatitidis* | [(42,43)](https://www.zotero.org/google-docs/?OcC3eO) |
| 143_P_08_S8 | PRJNA1201237 | *B. dermatitidis* | [(42,43)](https://www.zotero.org/google-docs/?Je10Vm) |
| Dr_Anuradha_Fungal_WGS_S13 | PRJNA1201237 | *B. dermatitidis* | [(42,43)](https://www.zotero.org/google-docs/?BuUnt6) |
| Ep_130_s_7 | PRJNA178178 | *Blastomyces* *sp.* | [(41)](https://www.zotero.org/google-docs/?CBTirl) |
| ep139_s_1 | PRJNA178178 | *Blastomyces* sp. | [(41)](https://www.zotero.org/google-docs/?CFJTKf) |
| Pb_339 | SRR4024750 | *Paracoccidioides restrepiensis* | (44) |
| Pb_60855 | SRR4024748 | *P. restrepiensis* | (44) |
| Pb_66_ATCACG_L001 | SAMN05171529 | *P. restrepiensis* | (44) |
| Pb_jam | SRR4024745 | *P. restrepiensis* | (44) |
| PbD02_TAGCTT_L001 | SRR4024744 | *P. restrepiensis* | (44) |
| Ep_9510_s_8 | PRJNA416769 | *P. restrepiensis* | [(28)](https://www.zotero.org/google-docs/?JfHDgi) |
| Ec_4076_s_7 | PRJNA178252 | *Emmonsia crescens* | [(41)](https://www.zotero.org/google-docs/?WTK3VV) |
| s_2 | PRJNA178252 | *E. crescens* | [(41)](https://www.zotero.org/google-docs/?ezDC0E) |

**TABLE S4.** Previous studies listing clinical characteristics of histoplasmosis caused by different phylogenetic species of *Histoplasma*.

| **Reference** | **Species included** |
| --- | --- |
| (34) | Mz5-like, *H. capsulatum ss*, Amazon I, Amazon II, *H. suramericanum* |
| [(30)](https://www.zotero.org/google-docs/?xdMLIn) | RJ |
| This study | *Africa, H. ohiense, H. mississippiense* |

**TABLE S5.** Loci under selection in both African lineages of *Histoplasma*, *Africa* and *Hcf*.

| **Lineage** | **Locus** | **Protein Name** | **PBE** |
| --- | --- | --- | --- |
| Hcf | MRP49 | 50S ribosomal protein Mrp49 | 1.14 |
| Hcf | WHI2 | stress response protein Whi2, partial | 1.19 |
| Hcf | PEX20 | peroxin 20, partial | 1.03 |
| Hcf | SEC13 | protein transporter SEC13 | 1.03 |
| Hcf | PIN4 | peptidyl-prolyl cis-trans isomerase pin4 | 1.30 |
| Hcf | CDC16 | cell division cycle protein | 1.06 |
| Hcf | ERG7 | oxidosqualene:lanosterol cyclase, partial | 1.10 |
| Hcf | RAB6 | GTP-binding protein ryh1, partial | 1.22 |
| Hcf | LYS1 | saccharopine dehydrogenase | 1.09 |
| Hcf | SEC2 | GDP/GTP exchange factor Sec2p | 1.04 |
| Hcf | SMD3 | small nuclear ribonucleoprotein Sm D3 | 1.17 |
| Hcf | RNH1 | RNase H domain-containing protein | 1.10 |
| Hcf | LSG1 | large subunit GTPase | 1.02 |
| Hcf | KCC4 | serine/threonine-protein kinase | 1.20 |
| Hcf | PRP18 | pre-mRNA-splicing factor | 1.36 |
| Hcf | GPI3 | phosphatidylinositol N-acetylglucosaminyltransferase GPI3 | 1.29 |
| Hcf | SEC65 | signal recognition particle SEC65 subunit | 1.06 |
| Hcf | PAN3 | PAB-dependent poly(A)-specific ribonuclease subunit pan3 | 0.97 |
| Hcf | FZO1 | transmembrane GTPase fzo1 | 1.16 |
| Hcf | RRM3 | DNA repair and recombination protein pif1 | 1.44 |
| Hcf | STE4 | small G-beta protein GPB, partial | 1.39 |
| Hcf | PHN1 | phosducin | 1.16 |
| Hcf | ATG16 | autophagy protein | 1.20 |
| Hcf | YPT7 | GTPase Rab7 | 1.56 |
| Hcf | SPB1 | AdoMet-dependent rRNA methyltransferase spb1 | 1.04 |
| Hcf | ALG3 | mannosyltransferase | 1.95 |
| Hcf | PKC1 | protein kinase | 1.72 |
| Hcf | GEA2 | Sec7 domain-containing protein | 1.36 |
| Hcf | MRPL19 | 60S ribosomal protein L19 | 1.01 |
| Hcf | SVF1 | Svf1 family protein | 1.01 |
| Hcf | RIO2 | serine/threonine-protein kinase RIO2 | 1.13 |
| Hcf | RPN1 | 26S proteasome regulatory subunit | 1.13 |
| Hcf | PPS1 | pps1 dual specificty phosphatase | 1.13 |
| Hcf | CPA2 | carbamoyl-phosphate synthase | 0.97 |
| Hcf | RPS15 | 40S ribosomal protein S15 | 1.19 |
| Hcf | CYS3 | cystathionine-gamma-lyase | 1.20 |
| Hcf | RPC25 | DNA-directed RNA polymerase III 25 kD polypeptide | 1.13 |
| Hcf | NOP12 | nucleolar protein 12, RNA binding protein | 1.49 |
| Hcf | DEP1 | transcriptional regulatory protein DEP1 | 1.38 |
| Hcf | STT3 | oligosaccharyl transferase stt3 subunit | 1.66 |
| Hcf | CSN8 | COP9 signalosome complex subunit 8 | 1.17 |
| Hcf | YOS9 | misfolded glycoproteins degradation protein Yos9 | 1.52 |
| Hcf | SLA1 | cytoskeleton assembly control protein SLA1p | 1.52 |
| Hcf | RCL1 | RNA-3\\'-phosphate cyclase | 1.40 |
| Hcf | NOC1 | Noc1p protein | 1.40 |
| Hcf | BRR2 | pre-mRNA-splicing factor brr2, oligopeptidase | 1.40 |
| Hcf | CSN1 | COP9 signalosome complex subunit | 1.01 |
| Hcf | MET17B | cysteine synthase | 1.00 |
| Hcf | APG6 | autophagy protein Apg6 | 1.42 |
| Hcf | PEX13 | peroxin 13 | 1.42 |
| Hcf | ERO1 | oxidoreductin | 1.15 |
| Hcf | RPB7 | RNA polymerase II subunit 7 | 1.54 |
| Hcf | SPC2 | signal peptidase complex component | 1.73 |
| Hcf | MMS19 | DNA repair/transcription protein | 2.12 |
| Hcf | NOC4 | CBF/Mak21 family | 1.56 |
| Hcf | ATP4 | ATP synthase subunit 4 | 1.56 |
| Hcf | TFG2 | transcription initiation factor iif | 0.99 |
| Hcf | SIN3 | transcriptional repressor Sin3p | 1.40 |
| Hcf | SKT5 | protoplast regeneration and killer toxin resistance protein | 1.45 |
| Hcf | CHS4 | chitin synthase | 1.09 |
| Hcf | SEC72 | F-box domain-containing protein | 1.16 |
| Hcf | SQT1 | ribosome assembly protein SQT1 | 1.05 |
| Hcf | SPS19 | oxidoreductase | 2.04 |
| Hcf | CCL1 | cyclin-dependent protein kinase regulator | 1.03 |
| Hcf | GCS1 | zinc finger protein | 1.37 |
| Hcf | MEF2 | elongation factor G | 1.85 |
| Hcf | VPS17 | vacuolar sorting-associated protein | 1.85 |
| Hcf | THO2 | tho2 protein | 1.08 |
| Hcf | CSN6 | COP9 complex subunit 6 | 1.94 |
| Hcf | CWC24 | pre-mRNA-splicing factor cwc24 | 1.15 |
| Hcf | ERF4 | ras modification protein, ERF4 superfamily | 1.15 |
| Hcf | MEK1 | serine/threonine-protein kinase Chk2/Mek1 | 1.07 |
| Hcf | URA7 | CTP synthase | 1.89 |
| Hcf | SAC3 | MCM3-associated protein | 1.09 |
| Hcf | FIG4 | polyphosphoinositide phosphatase Fig4 | 1.23 |
| Hcf | SEC18 | secretory protein nsfA | 1.39 |
| Hcf | GST1c | glutathione transferase | 1.24 |
| Hcf | TAZ1 | tafazzin | 1.01 |
| Hcf | ESF2 | pre-rRNA-processing protein esf2 | 1.07 |
| Hcf | BST1 | GPI inositol-deacylase | 1.06 |
| Hcf | COX11 | mitochondrial cytochrome c oxidase assembly factor | 1.16 |
| Hcf | HCL1 | hydroxymethylglutaryl-CoA lyase | 1.03 |
| Hcf | ARG8 | acetylornithine aminotransferase | 1.05 |
| Hcf | PRP8 | mRNA splicing protein PRP8 | 1.65 |
| Hcf | RPL34 | 60S ribosomal protein L34B | 1.12 |
| Hcf | HMT1 | heavy metal tolerance protein | 1.25 |
| Hcf | SLX4 | structure-specific endonuclease subunit SLX4 | 1.12 |
| Hcf | SRW1 | cell division cycle protein | 1.65 |
| Hcf | PDX1 | pyruvate dehydrogenase complex component Pdx1 | 1.65 |
| Hcf | SMC1 | structural maintenance of chromosomes protein 1 | 1.05 |
| Hcf | SFK1 | FK506 suppressor Sfk1 | 0.99 |
| Hcf | TOF1 | topoisomerase 1 | 1.02 |
| Hcf | ARP9 | actin-like protein arp9 | 1.02 |
| Hcf | NSE1 | DNA repair protein Nse1 | 1.07 |
| Hcf | YPT1 | GTP-binding protein ypt1 | 1.19 |
| Hcf | SKI2 | translation repressor | 1.28 |
| Hcf | CHS2 | class II chitin synthase | 1.27 |
| Africa | STR1 | WD domain-containing protein | 2.53 |
| Africa | RAD14 | DNA repair protein rad14 | 2.69 |
| Africa | GCD10 | tRNA(m1A58)-methyltransferase subunit trm6 | 1.63 |
| Africa | CBP1 | calcium-binding protein | 1.64 |
| Africa | HSF1 | heat shock transcription factor | 1.56 |
| Africa | TYR5 | tyrosinase | 1.49 |
| Africa | TFA2 | transcription initiation factor IIE subunit beta | 1.65 |
| Africa | GAA1 | rhomboid protein | 1.96 |
| Africa | NIF3 | NGG1 interacting factor Nif3 | 1.84 |
| Africa | VPS64 | cytoplasm to vacuole targeting Vps64 | 1.49 |
| Africa | SHM2 | serine hydroxymethyltransferase, partial | 1.66 |
| Africa | ENG6 | 2,3-bisphosphoglycerate-independent phosphoglycerate mutase | 1.66 |
| Africa | UTP6 | U3 snoRNP protein | 2.05 |
| Africa | AUR1 | aureobasidin resistance protein Aur1 | 1.52 |
| Africa | BUD5 | ras guanine-nucleotide exchange protein Cdc25p | 1.65 |
| Africa | SAC6 | fimbrin, partial | 1.81 |
| Africa | AIM31 | mitochondrial hypoxia responsive domain-containing protein | 2.27 |
| Africa | PIN4 | peptidyl-prolyl cis-trans isomerase pin4 | 1.44 |
| Africa | URH1 | uridine nucleosidase | 1.68 |
| Africa | PUT1b | proline oxidase | 1.84 |
| Africa | EXO2 | exonuclease | 2.02 |
| Africa | CNA1 | serine/threonine phosphatase 2B catalytic subunit | 1.99 |
| Africa | TYR7 | tyrosinase | 1.70 |
| Africa | TRM12 | tRNA wybutosine-synthesizing protein | 2.20 |
| Africa | SSP120 | secretory pathway protein Ssp120 | 2.20 |
| Africa | VPS15 | VPS15 protein kinase | 3.03 |
| Africa | DBP10 | ATP-dependent RNA helicase DBP10 | 1.70 |
| Africa | PPO3 | heme peroxidase | 2.40 |
| Africa | SEC2 | GDP/GTP exchange factor Sec2p | 1.67 |
| Africa | SAP114 | splicing factor 3 subunit 1 | 1.53 |
| Africa | CRB3 | WD domain-containing protein | 1.54 |
| Africa | MDL1 | ATP-dependent permease MDL1, partial | 2.52 |
| Africa | ERB1 | eukaryotic ribosome biogenesis protein | 1.46 |
| Africa | END3 | cytoskeletal adaptor protein SagA | 1.61 |
| Africa | PUS7 | pseudouridine synthase | 1.54 |
| Africa | ALO1 | D-arabinono-1,4-lactone oxidase | 1.78 |
| Africa | ECM33 | GPI-anchored cell wall organization protein Ecm33 | 3.23 |
| Africa | RPS5 | 40S ribosomal protein S5A | 1.54 |
| Africa | NOP58 | nucleolar protein NOP58 | 1.55 |
| Africa | TRS31 | BET3 family protein | 1.56 |
| Africa | PUS4 | pseudouridine synthase | 1.87 |
| Africa | MET17B | cysteine synthase | 2.30 |
| Africa | APG6 | autophagy protein Apg6 | 2.97 |
| Africa | SEC3 | exocyst complex component Sec3 | 1.73 |
| Africa | GYP10 | GTPase activating protein gyp10, partial | 2.12 |
| Africa | CON132 | conidiation-specific protein 13, 2nd best hit | 1.52 |
| Africa | COX15 | cytochrome c oxidase assembly protein | 1.69 |
| Africa | KRS1 | lysyl-tRNA synthetase | 1.64 |
| Africa | MTP1 | glycerophosphoinositol permease | 1.48 |
| Africa | ATP11 | F1 ATPase assembly protein | 1.85 |
| Africa | PRE3 | proteasome component | 1.59 |
| Africa | PAS1 | peroxin 1 | 1.59 |
| Africa | MBP1 | macrophage binding protein | 1.60 |
| Africa | RAD3 | DNA repair helicase RAD3 | 1.64 |
| Africa | IST1 | DUF292 domain-containing protein | 1.64 |
| Africa | ATG27 | autophagy protein | 1.68 |
| Africa | NIT9 | C2 domain-containing protein, nitrosative stress-induced transcript, conidia-enriched transcript | 1.69 |
| Africa | NIT37 | nitrositive-stress induced transcript | 1.69 |
| Africa | INV1 | involucrin repeat protein | 2.13 |
| Africa | HIR1 | histone transcription regulator slm9 | 1.85 |
| Africa | RAS2 | GTPase Ras2p | 1.50 |
| Africa | WHC1 | white collar 1 | 1.50 |
| Africa | SGD1 | nuclear protein | 1.51 |
| Africa | PRP24 | RNA-binding protein Prp24 | 1.48 |
| Africa | SNF1 | carbon catabolite derepressing protein kinase | 1.44 |
| Africa | SEC7 | protein transporter SEC7 | 1.61 |
| Africa | LAT1 | dihydrolipoamide S-acetyltransferase | 1.61 |
| Africa | MRPL31 | mitochondrial 54S ribosomal protein YmL31 | 1.92 |
| Africa | DBP9 | ATP-dependent RNA helicase DBP9 | 2.30 |
| Africa | RPS13 | 40S ribosomal protein S13 | 2.81 |
| Africa | RPD3 | histone deacetylase | 1.54 |
| Africa | TIM50 | import inner membrane translocase subunit tim-50 | 1.69 |
| Africa | ABC4 | ABC transporter CDR4 | 1.78 |
| Africa | PIC2 | mitochondrial phosphate carrier protein | 1.53 |
| Africa | TYR2 | polyphenoloxidase | 1.76 |
| Africa | ECM29 | proteasome component ECM29 | 1.79 |
| Africa | CRC1 | carnitine/acyl carnitine carrier | 1.56 |
| Africa | NCS1 | calcium sensor protein | 1.84 |
| Africa | TOP2 | DNA topoisomerase II | 1.57 |
| Africa | LYS7 | superoxide dismutase 1 copper chaperone | 1.76 |
| Africa | ABP140 | actin filament binding protein | 1.62 |
| Africa | TFB2 | RNA pol II transcription initiation subunit | 1.51 |
| Africa | CATB | catalase B | 1.53 |
| Africa | TRI7 | TRI7-like toxin biosynthesis protein | 1.50 |
| Africa | BRR6 | nuclear membrane protein | 1.50 |
| Africa | RVB2 | RuvB-like helicase | 1.81 |
| Africa | UFD1 | ubiquitin fusion degradation protein | 2.76 |
| Africa | TUB1 | tubulin alpha-1 subunit | 1.59 |
| Africa | FLX1 | folate carrier protein | 2.56 |
| Africa | FOX2 | acetoacetyl-CoA reductase | 1.47 |
| Africa | CAC2 | chromatin assembly factor 1 subunit B | 1.67 |
| Africa | bimC | kinesin-like protein bimC | 1.68 |

**TABLE S6**. D-statistics support a complex history of divergence and gene flow in African *Histoplasma*.
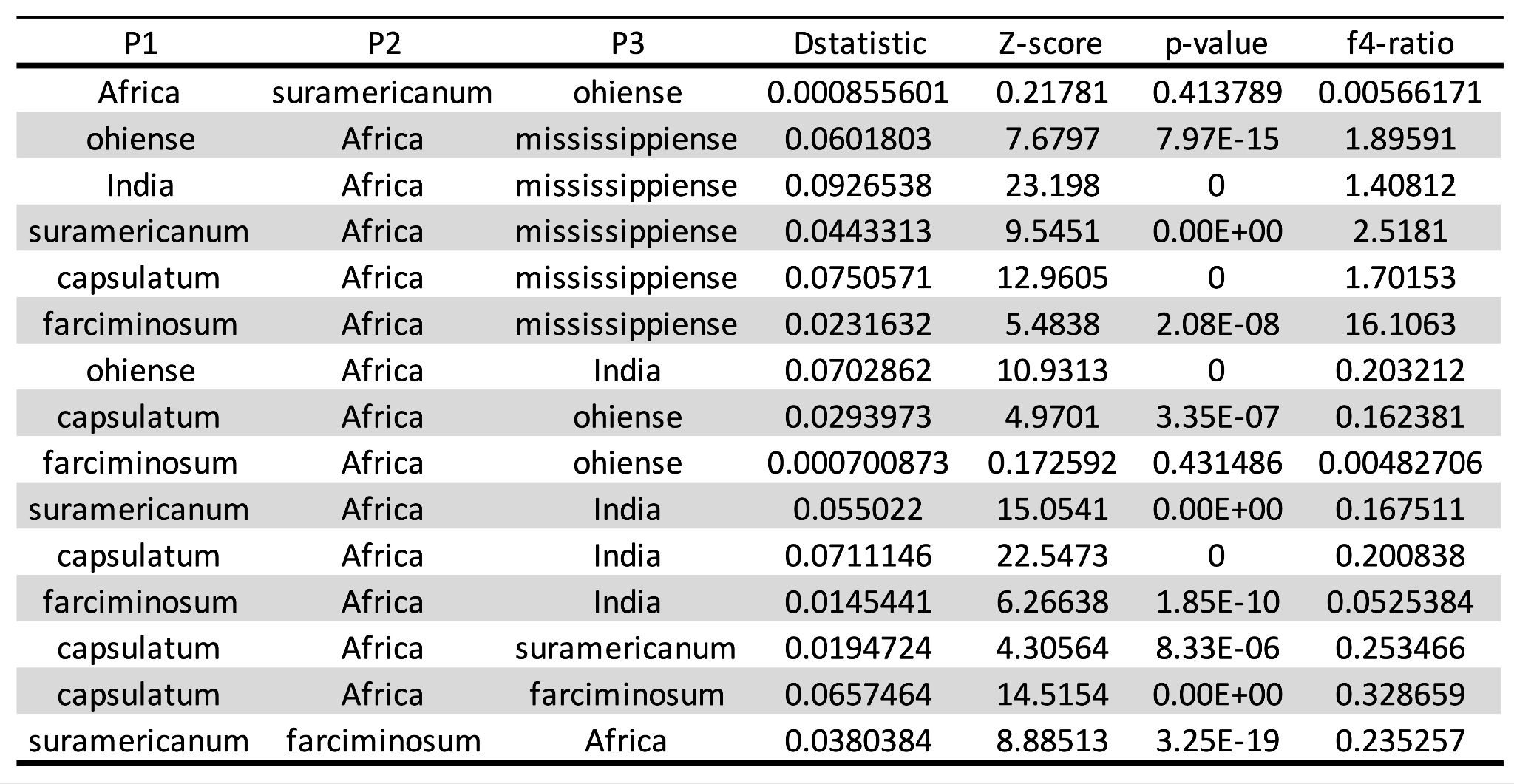


**TABLE S7.** Comparisons between the proportion of cases showing skin involvement between pairs of *Histoplasma* phylogenetic species. We used a 2-sample test for equality of proportions with continuity correction. The numbers in the ‘Proportion’ columns show the number of patients with skin symptoms; the denominator shows the total number of cases for which we have information about skin involvement. We adjusted the P-values for multiple comparisons using the Holm method. We only report the pairwise comparisons involving the *Africa* lineage as they are the ones relevant for this study. Power for the proportion test was calculated using the smallest denominator of the two proportions.

| **Species 1** | **Proportion 1** | **Species 2** | **Proportion 2** | **Proportion test** | | |
| --- | --- | --- | --- | --- | --- | --- |
|  |  |  |  | **X2** | **P** | **pwr** |
| *Africa* | 9/18 | *H. capsulatum ss* | 0/12 | 6.356 | 0.035 | 0.856 |
| *Africa* | 9/18 | *H. suramericanum* | 2/8 | 0.579 | 0.447 | 0.169 |
| *Africa* | 9/18 | mz5-line | 0/42 | 20.940 | < 0.001 | 0.168 |
| *Africa* | 9/18 | *H. mississippiense* | 1/9 | 2.402 | 0.242 | 0.426 |
| *Africa* | 9/18 | *H. ohiense* | 0/19 | 7.249 | 0.028 | 0.967 |

**TABLE S8.** Comparisons between the proportion of cases in HIV patients between pairs of *Histoplasma* phylogenetic species. We used a 2-sample test for equality of proportions with continuity correction. The numerator in the ‘Proportion’ columns shows the number of HIV+ patients; the denominator shows the total number of cases for which we have information of HIV status. We adjusted the P-values for multiple comparisons using the Holm method. We only report the pairwise comparisons involving the *Africa* lineage as they are the ones relevant for this study. Power for the proportion test was calculated using the smallest denominator of the two proportions.

| **Species 1** | **Proportion 1** | **Species 2** | **Proportion 2** | **Proportion test** | | |
| --- | --- | --- | --- | --- | --- | --- |
|  |  |  |  | **X^2^** | **P** | **pwr** |
| *Africa* | 15/16 | *H. capsulatum ss* | 15/15 | < 0.01 | 1.000 | 0.161 |
| *Africa* | 15/16 | *H. suramericanum* | 14/14 | < 0.01 | 1.000 | 0.152 |
| *Africa* | 15/16 | mz5-line | 44/45 | < 0.01 | 1.000 | 0.978 |
| *Africa* | 15/16 | *H. mississippiense* | 3/9 | 7.648 | 0.028 | 0.817 |
| *Africa* | 15/16 | *H. ohiense* | 10/17 | 3.738 | 0.213 | 0.655 |

**TABLE S9.** Comparisons between the proportion of cases per sex between pairs of *Histoplasma* phylogenetic species. We used a 2-sample test for equality of proportions with continuity correction. The numbers in the ‘Proportion’ columns show the number of female patients; the denominator shows the total number of cases for which we have information about sex. We adjusted the P-values for multiple comparisons using the Holm method. We only report the pairwise comparisons involving the *Africa* lineage as they are the ones relevant for this study. Power for the proportion test was calculated using the smallest denominator of the two proportions.

| **Species1** | **Proportion 1** | **Species 2** | **Proportion 2** | **Proportion test** | | |
| --- | --- | --- | --- | --- | --- | --- |
|  |  |  |  | **X^2^** | **P** | **pwr** |
| *Africa* | 9/18 | *H. capsulatum ss* | 1/15 | 5.367 | 0.103 | 0.712 |
| *Africa* | 9/18 | *H. suramericanum* | 2/8 | 0.579 | 0.997 | 0.222 |
| *Africa* | 9/18 | mz5-line | 15/45 | 0.890 | 0.997 | 0.169 |
| *Africa* | 9/18 | *H. mississippiense* | 2/9 | 0.940 | 0.997 | 0.222 |
| *Africa* | 9/18 | *H. ohiense* | 4/18 | 1.926 | 0.661 | 0.222 |

**TABLE S10.** Tukey test pairwise comparisons between median age at diagnosis between patients with histoplasmosis caused by six different phylogenetic species of *Histoplasma*.

| **Species 1** | **Species 2** | **Estimate** | **Std. Error** | **t-value** | **P** |
| --- | --- | --- | --- | --- | --- |
| LAmB | Amazon_III | 6.000 | 16.115 | 0.372 | 0.999 |
| *Africa* | Amazon_III | 17.118 | 7.484 | 2.287 | 0.322 |
| Amazon_II | Amazon_III | 21.500 | 12.308 | 1.747 | 0.680 |
| *H. capsulatum* | Amazon_III | 17.083 | 7.830 | 2.182 | 0.385 |
| *H. suramericanum* | Amazon_III | 14.750 | 8.386 | 1.759 | 0.672 |
| mz5-like | Amazon_III | 22.925 | 6.978 | 3.285 | 0.030 |
| *H. mississippiense* | Amazon_III | 30.667 | 8.205 | 3.737 | 0.008 |
| *H. ohiense* | Amazon_III | 22.579 | 7.39396 | 3.054 | 0.057 |
| *Africa* | LAmB | 11.117 | 15.13719 | 0.734 | 0.998 |
| Amazon_II | LAmB | 15.5 | 18.01685 | 0.86 | 0.993 |
| *H. capsulatum* | LAmB | 11.083 | 15.31138 | 0.724 | 0.998 |
| *H. suramericanum* | LAmB | 8.75 | 15.60305 | 0.561 | 0.999 |
| mz5-like | LAmB | 16.925 | 14.89345 | 1.136 | 0.960 |
| *H. mississippiense* | LAmB | 24.667 | 15.50644 | 1.591 | 0.778 |
| *H. ohiense* | LAmB | 16.579 | 15.09286 | 1.098 | 0.967 |
| Amazon_II | *Africa* | 4.382 | 10.99691 | 0.399 | 0.999 |
| *H. capsulatum* | *Africa* | -0.034 | 5.54648 | -0.006 | 1.000 |
| *H. suramericanum* | *Africa* | -2.368 | 6.30716 | -0.375 | 0.999 |
| mz5-like | *Africa* | 5.807 | 4.259 | 1.364 | 0.891 |
| *H. mississippiense* | *Africa* | 13.549 | 6.064 | 2.234 | 0.352 |
| *H. ohiense* | *Africa* | 5.461 | 4.911 | 1.112 | 0.965 |
| *H. capsulatum* | Amazon_II | -4.417 | 11.235 | -0.393 | 0.999 |
| *H. suramericanum* | Amazon_II | -6.75 | 11.630 | -0.58 | 0.999 |
| mz5-like | Amazon_II | 1.425 | 10.659 | 0.134 | 1.000 |
| *H. mississippiense* | Amazon_II | 9.167 | 11.500 | 0.797 | 0.995 |
| *H. ohiense* | Amazon_II | 1.079 | 10.936 | 0.099 | 1.000 |
| *H. suramericanum* | *H. capsulatum* | -2.333 | 6.715 | -0.348 | 0.999 |
| mz5-like | *H. capsulatum* | 5.842 | 4.841 | 1.206 | 0.943 |
| *H. mississippiense* | *H. capsulatum* | 13.583 | 6.487 | 2.094 | 0.441 |
| *H. ohiense* | *H. capsulatum* | 5.496 | 5.423 | 1.013 | 0.980 |
| mz5-like | *H. suramericanum* | 8.175 | 5.697 | 1.435 | 0.861 |
| *H. mississippiense* | *H. suramericanum* | 15.917 | 7.148 | 2.227 | 0.357 |
| *H. ohiense* | *H. suramericanum* | 7.829 | 6.200 | 1.263 | 0.927 |
| *H. mississippiense* | mz5-like | 7.742 | 5.427 | 1.426 | 0.865 |
| *H. ohiense* | mz5-like | -0.346 | 4.099 | -0.084 | 1.000 |
| *H. ohiense* | *H. mississippiense* | -8.088 | 5.953 | -1.359 | 0.893 |

**TABLE S11.** Summary of linear models of climate predictors of histoplasmosis caseload.

| **Climate Variable** | **Slope** | **Intercept** | ***F*-statistic** | ***P*-Value** |
| --- | --- | --- | --- | --- |
| Hypervolume | 9.715 ± 4.781 | -18.119 ± 15.835 | *F*_1,29_ = 4.129 | 0.051 |
| Bio1 mean  min  max | -0.443 ± 1.439  -0.414 ± 0.654  -0.998 ± 2.165 | 23.761 ± 35.194  20.115 ± 12.040  40.741 ± 60.309 | *F*_1,29_ = 0.095  *F*_1,29_ = 0.400  *F*_1,29_ = 0.213 | 0.760  0.532  0.648 |
| Bio2 mean  min  max | -0.156 ± 2.209  -2.094 ± 2.620  1.538 ± 1.931 | 14.875 ± 26.978  29.454 ± 21.020  -10.474 ± 29.768 | *F*_1,29_ = 0.005  *F*_1,29_ = 0.639  *F*_1,29_ = 0.635 | 0.944  0.431  0.432 |
| Bio5 mean  min  max | -0.306 ± 1.137  -0.519 ± 0.671  0.027 ± 1.092 | 23.362 ± 38.724  26.182 ± 17.559  11.969 ± 42.632 | *F*_1,29_ = 0.072  *F*_1,29_ = 0.599  *F*_1,29_ = 0.001 | 0.790  0.445  0.981 |
| Bio6 mean  min  max | -0.232 ± 0.929  -0.209 ± 0.617  0.075 ± 1.366 | 16.358 ± 14.093  14.538 ± 6.254  11.530 ± 27.257 | *F*_1,29_ = 0.063  *F*_1,29_ = 0.115  *F*_1,29_ = 0.003 | 0.804  0.737  0.957 |
| Bio12 mean  min  max | 0.0008 ± 0.0071  -0.006 ± 0.009  0.005 ± 0.004 | 12.149 ± 8.526  16.170 ± 6.218  3.222 ± 9.612 | *F*_1,29_ = 0.013  *F*_1,29_ = 0.492  *F*_1,29_ = 1.281 | 0.909  0.489  0.267 |

**Table S12.** Summary of 17 bioclimatic variables, summarized across 31 sampled countries shows variation in climatic niche breadth among countries.

| **Clim Variable** | **Mean** | **Minimum** | **Maximum** | **Coeff Variation** |
| --- | --- | --- | --- | --- |
| bio1_range | 6.2264 | 1.3917 | 15.504 | 55.6813 |
| bio2_range | 5.4334 | 1.6396 | 8.4858 | 28.9998 |
| bio4_range | 200.1499 | 21.8749 | 581.2543 | 72.8968 |
| bio5_range | 8.3886 | 3.4 | 16.11 | 35.7706 |
| bio6_range | 8.5042 | 3 | 16.9025 | 45.1285 |
| bio8_range | 7.4019 | 0.9333 | 21.1137 | 63.3472 |
| bio9_range | 7.7312 | 1.9667 | 15.7696 | 53.87 |
| bio10_range | 7.0549 | 2.2092 | 17.4058 | 47.7981 |
| bio11_range | 6.8974 | 1.2667 | 15.9671 | 59.5516 |
| bio12_range | 1037.959 | 112 | 2175.5 | 52.1228 |
| bio13_range | 214.5935 | 28 | 730.8 | 60.3801 |
| bio14_range | 30.379 | 0 | 114.025 | 105.4446 |
| bio15_range | 61.1099 | 11.0469 | 133.407 | 48.5954 |
| bio16_range | 527.0516 | 72 | 1630.6 | 58.6986 |
| bio17_range | 115.2129 | 1 | 393 | 92.584 |
| bio18_range | 300.1589 | 3 | 842.8 | 52.0763 |
| bio19_range | 510.9 | 6 | 2389.4 | 98.0398 |

**TABLE S13.** Current climatic suitability for histoplasmosis and projections of future suitability under three regimes of warming over three time intervals.

|  | Current Conditions | ssp126 2021-2040 | ssp126 2041-2060 | ssp126 2061-2080 | ssp370 2021-2040 | ssp370 2041-2060 | ssp370 2061-2080 | ssp585 2021-2040 | ssp585 2041-2060 | ssp585 2061-2080 |
| --- | --- | --- | --- | --- | --- | --- | --- | --- | --- | --- |
| Mean Habitat Suitability | 15.76597 ± 0.0148 | 23.085 ± 0.0187 | 22.2228 ± 0.0183 | 21.9163 ± 0.018 | 22.7921 ± 0.0185 | 19.8577 ± 0.0169 | 17.285 ± 0.0155 | 22.7354 ± 0.0185 | 19.678 ± 0.0170 | 15.766 ± 0.0148 |
| Median Habitat Suitability | 2.331 | 4.7473 | 4.314 | 4.2822 | 4.638 | 3.549 | 2.9293 | 4.6834 | 3.4555 | 2.331 |
| Percent < 0.4 | 10.393 | 15.583 | 15.313 | 15.493 | 15.714 | 14.684 | 12.021 | 15.328 | 13.726 | 10.393 |
| Percent between 0.4 and 0.6 | 83.130 | 68.798 | 70.519 | 70.993 | 69.252 | 74.787 | 80.460 | 69.630 | 75.657 | 83.130 |
| Percent > 0.6 | 6.477 | 15.619 | 14.168 | 13.515 | 15.034 | 10.530 | 7.519 | 15.042 | 10.617 | 6.477 |

**SUPPLEMENTARY FIGURES**

**FIGURE S1.** **ASTRAL tree from gene genealogies generated from genomic windows.** The numbers at each node represent the bootstrap support and the CF. Note that the lineages present in Africa (Africa, *Hcf*, mz5-like) have high support.

**FIGURE S2. BUSCO-based concordance analyses show similar results to those obtained based on genome windows.** The numbers at each node represent the bootstrap support and the CF. Note that the two lineages present in Africa (Africa, Hcf, mz5-like) have high support.

**FIGURE S3. Phylogenetic relationships in Histoplasma using a different reference genome.** Topologies using a different reference genome (H88) vary in the reciprocal relationships between species, but in all cases the *Africa* and *Hcf* lineages form monophyletic groups.

**FIGURE S4. π along the *Hcf* genome.**

**FIGURE S5**. **Dxy along the genome between *Africa* and *Hcf*.**

**FIGURE S6. *Hcf* colony and microscopy morphology. A.** Colony morphology at 25ºC. **B.** Colony morphology at 37ºC. **C.** Light microscopy with lactophenol blue stain showing the mycelial stage of Histoplasma Hcf. **D and E.** Electron microscopy of a 2 week culture at 37ºC showing the *Histoplasma* yeast form. **Panels** A-C correspond to SA19VMG; Panels D and E correspond to SA20VMK.

**FIGURE S7. The distributions of climatic suitability in the sampled region shift over time in three models of warming.** A MaxENT model trained on the climatic conditions of the African countries with the three highest histoplasmosis caseloads showed broad climatic suitability for infection (blue violin). When applied to three models of climatic warming (yellow, orange, and red violins) across three time periods, the model predicts an ongoing human health threat from *Histoplasma*.


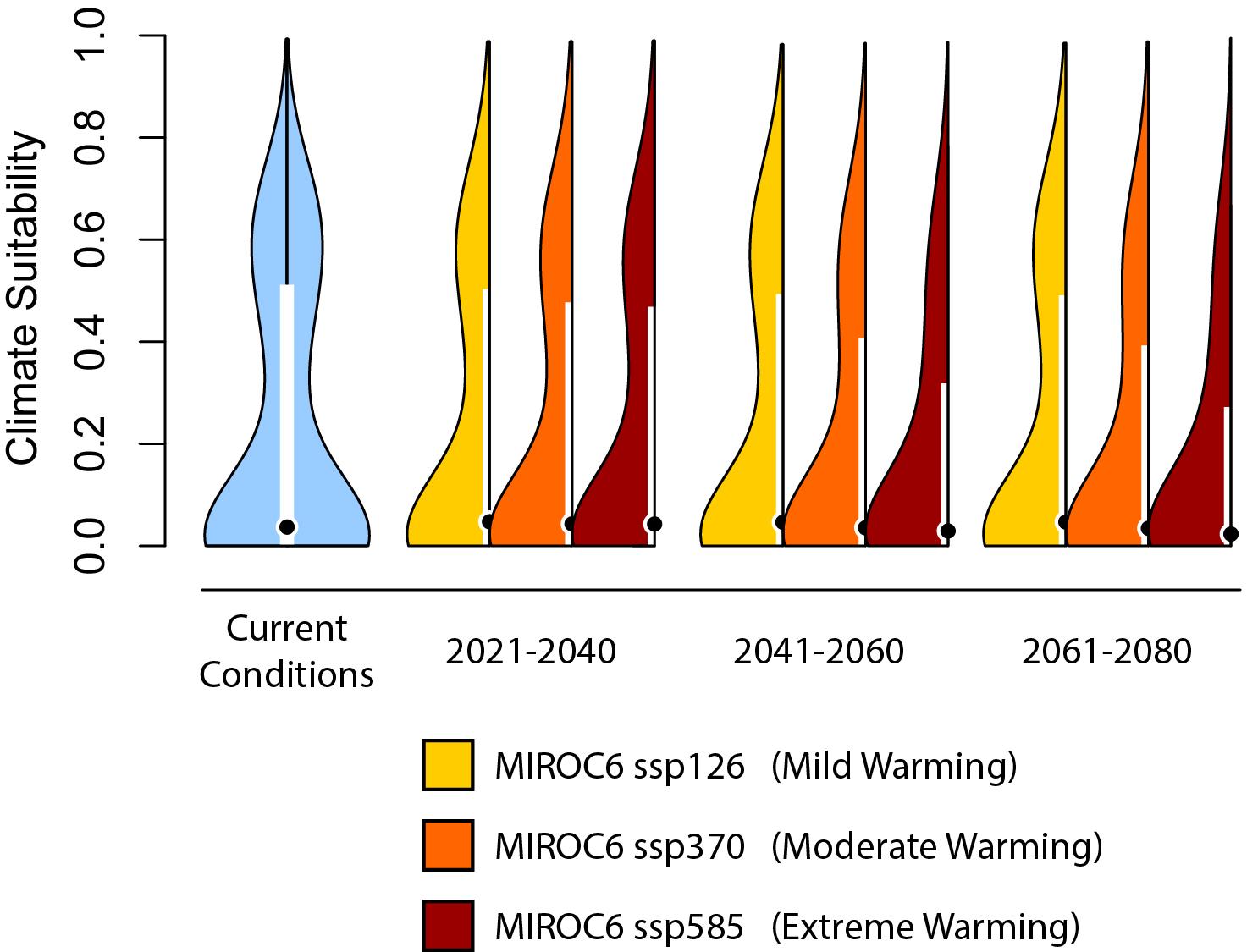


**FIGURE S8. Current bioclimatic conditions are broadly suitable to permit histoplasmosis infections across most of the African continent.** Regions in yellow have high climatic suitability. We used projections of climatic suitability via three models of climatic warming to visualize future climatic suitability, and how that suitability might shift across the continent in response to future warming.

**
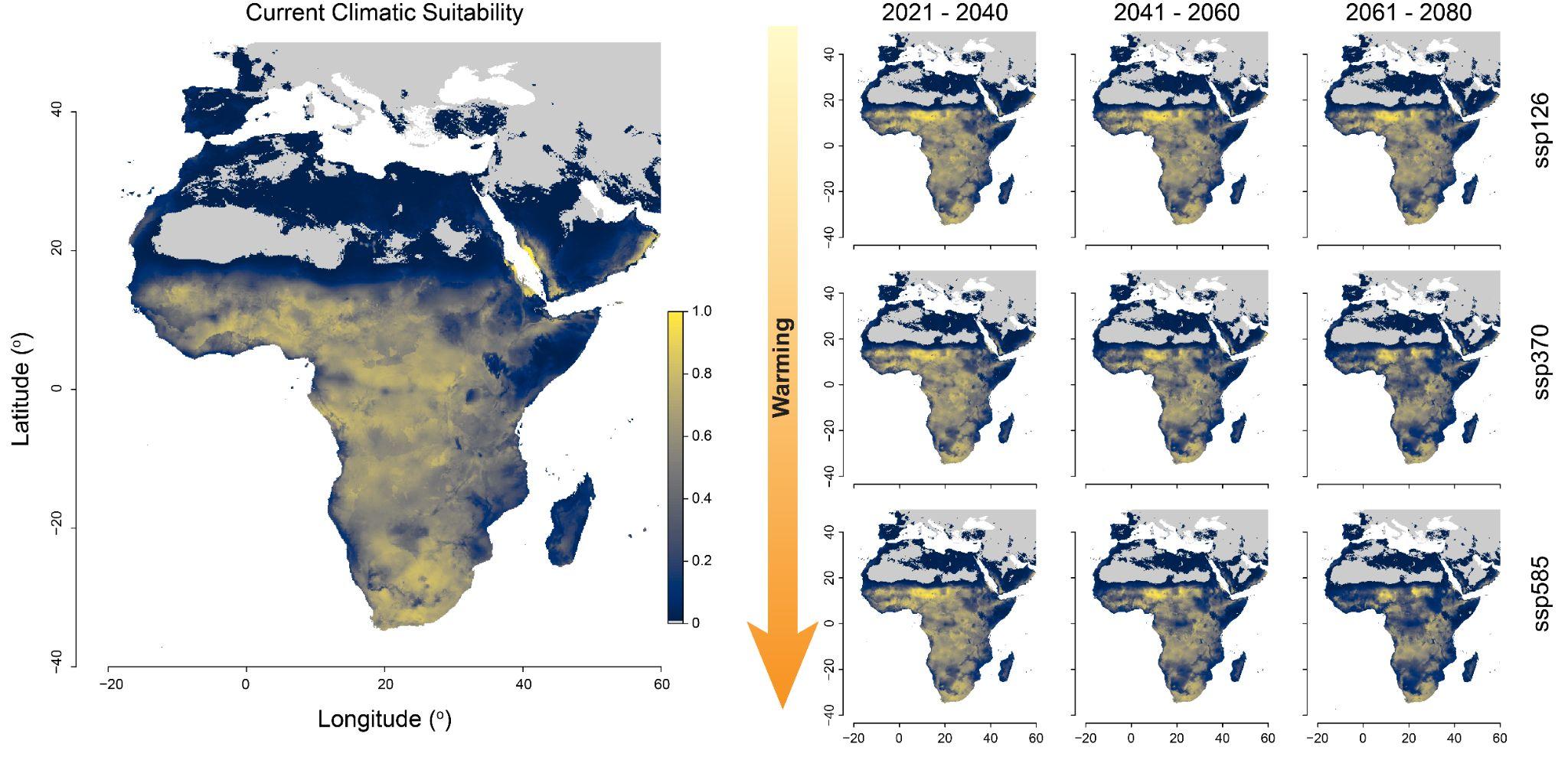
**
